# Ultrasound-Assisted Enzymatic Deamidation Treatment for Developing Soft Surimi Gel for Dysphagia: Gel Properties, In Vitro Digestibility, and Protein Mechanisms

**DOI:** 10.3390/foods15173036

**Published:** 2026-08-28

**Authors:** Wei Wang, Qing Shao, Lifei Wang, Shumin Yi, Xuepeng Li, Jianrong Li, Beibei Ye, Chang Zhang, Yongxia Xu, Wenhui Zhu

**Affiliations:** National & Local Joint Engineering Research Center of Storage, Processing and Safety Control Technology for Fresh Agricultural and Aquatic Products, College of Food Science and Engineering, Bohai University, Jinzhou 121013, China; wwll812002@163.com (W.W.); shaoqing1939827103@163.com (Q.S.); 18641692602@163.com (L.W.); xuepengli8234@163.com (X.L.); 13591278928@163.com (J.L.); beibeiy1993@163.com (B.Y.); zchangzc@163.com (C.Z.); xuyx1009@126.com (Y.X.); wenhuiby130@163.com (W.Z.)

**Keywords:** surimi gel, dysphagia, ultrasound-enzyme synergistic, vitro digestibility, protein mechanisms, gel properties

## Abstract

An urgent necessity exists to create foods that cater to the requirements of patients with dysphagia. The softened surimi gel appropriate for dysphagia patients was produced through ultrasonic-assisted Protein-glutaminase (PG) deamidation treatment. In comparison to the control group, the hardness and gel strength of softened surimi gel with a 0.2% PG addition diminished from 607.40 g and 530.79 g mm to 244.60 g and 78.61 g mm, respectively, satisfying the IDDSI Level 5 classification criteria (*p* < 0.05). The incorporation of PG transformed free water in the fish paste gel into less mobile water, markedly improving the gel’s water holding capacity. The ultrasound-assisted enzymatic deamidation treatment made the surimi gel produce more small molecular peptides and free amino acids during digestion. This treatment diminished hydrophobic interactions in myofibrillar protein gel, augmented hydrogen bonding, elevated the α-helix structure to 46.27%, and decreased the β-sheet structure to 19.18%, with significant differences compared to the control group (*p* < 0.05). The polarity of the tryptophan residue microenvironment alters and impeding the further folding of unfolded protein structures during heat-induced gelation. This study offers a fundamental theoretical framework for the development of specialized foods that possess safe swallowing and nutritional attributes.

## 1. Introduction

Contemporary research initiatives aimed at addressing dysphagia have focused on texture modification across the entire food matrix. For liquid foods, thickening is a common strategy to reduce bolus transit velocity in the pharynx and esophagus, whereas for solid and semi-solid foods, various processing techniques—including microwave, irradiation, and ultrasound—have been employed to modify food texture to comply with the International Dysphagia Diet Standardisation Initiative (IDDSI) framework. The velocity of food transit in the pharynx and esophagus diminishes with an increase in food viscosity, thereby adhering to the guidelines established by the International Dysphagia Diet Standardisation Initiative (IDDSI). Polysaccharides are frequently utilized as thickeners in the formulation of foods designed for dysphagia to enhance the viscosity of liquid foods [1,2,3]. Furthermore, diverse methods, including microwave, irradiation, and ultrasound, have been employed to alter the texture of food in order to produce IDDSI compliant food products [4,5].

The main aim of human consumption is to fulfill physiological nutritional needs and provide sensory pleasure. Nonetheless, impaired functionality of the swallowing organs markedly reduces the sensory experience for individuals suffering from dysphagia. The commercially available dysphagia foods currently on the market are generally deficient in several key areas. These issues encompass low nutritional density, subpar sensory attributes (regarding appearance, flavor, taste, and monotony), inadequate texture stability, limited variety, and elevated costs. The creation of foods that concurrently promote nutritional health and enable manageable dysphagia using natural ingredients constitutes the ideal resolution to the issue of dysphagia [6]. While sensory quality and clinical swallowing safety are ultimately critical for practical translation, the present study focuses on the foundational aspects of textural modification and in vitro digestibility assessment. The IDDSI framework testing employed herein serves as a preliminary instrumental screening tool for evaluating swallowing-suitable textures, rather than a substitute for formal sensory evaluation or clinical swallowing assessments (e.g., videofluoroscopic swallowing study or fiberoptic endoscopic evaluation). Such translational validations are beyond the scope of this initial investigation and will be addressed in subsequent studies. Recent research has aimed to adapt traditional foods to comply with the IDDSI standards in order to address the dietary requirements of individuals with dysphagia [7]. The application of sodium alginate has been shown to improve the hydrophilic interactions of composite gels derived from Antarctic krill and white shrimp, consequently decreasing gel hardness [8]. Fish flesh is abundant in high-quality proteins, vitamins, minerals, and other constituents that are readily assimilated and metabolized by the human body. Soft gels derived from surimi products designed to comply with the IDDSI framework will constitute a nutrient-dense dysphagia food. Research indicates that alkaline protease treatment of surimi gel can significantly enhance proteolysis, leading to gel softening [9]. The pH responsiveness of betaine allows the betaine, gelatin, and nanochitin complex to diminish interaction strength and create dysphagia food incorporating surimi protein [10].

The textural characteristics of surimi gels are significantly influenced by the stability of the cross-linking network of myofibrillar proteins. Regulating the charge distribution and conformation of myosin enables the controllable attenuation of the gel network, resulting in a softened gel appropriate for ingestion while preserving the protein’s structural integrity. The treatment of chicken breast myofibrillar protein with Protein-glutaminase (PG) enzyme markedly enhanced the net charge of the protein while preserving the composition of the majority of amino acids and protein subunits [11]. Besides enhancing protein solubility, emulsification, and foaming, PG deamidation treatment can alter food flavor [12]. Moderate deamidation has been reported to enhance certain gel characteristics of myofibrillar protein, particularly water retention and elasticity. However, for dysphagia-oriented applications, a critical balance must be achieved: hardness should be sufficiently reduced to minimize chewing effort and meet the IDDSI Level 5 criteria, while cohesiveness must be retained to prevent premature bolus disintegration during oral transit. Moreover, enhanced water-holding capacity is desirable not only for improving juiciness and palatability but also for ensuring adequate bolus moistness, which facilitates cohesive bolus formation and reduces pharyngeal residue. Therefore, PG deamidation is exploited in this study to achieve this balance by weakening excessive hydrophobic interactions (which primarily contribute to gel hardness) while reinforcing hydrogen bonding and electrostatic repulsion (which help retain water and maintain structural integrity without over-strengthening the gel network). The deamidation of the PG induces protein unfolding, leading to conformational alterations and protein denaturation, which exposes previously concealed hydrophobic regions. Furthermore, deamidation transforms amide groups into carboxyl groups, which markedly diminishes intramolecular and intermolecular hydrogen bonds and amplifies electrostatic repulsion among protein molecules, consequently enhancing solubility [13].

Investigations into specialized foods for individuals with dysphagia have concentrated on texture formulation and the assessment of swallowing safety [10,14]. The elderly, a significant demographic with dysphagia, typically experience physiological alterations, including diminished salivary secretion, impaired gastrointestinal motility, and variations in the electrolyte composition of digestive fluids. These factors collectively result in diminished efficiency of nutrient digestion and absorption. Novel foods should consider nutrient availability [15].

The International Dysphagia Diet Standardisation Initiative (IDDSI) framework classifies foods into eight levels (0–7) based on their textural properties. Among these, Level 5 (Minced & Moist) was selected as the target endpoint for this study for several reasons. First, Level 5 is appropriate for individuals with moderate to severe dysphagia who have reduced chewing ability but retain some tongue propulsion, representing a large segment of the target population. Second, this level requires foods that are soft, moist, and easily mashed with a fork, yet still form a cohesive bolus without separation of liquid and solid phases—characteristics that align well with the innate fibrous structure of surimi gels. Third, achieving Level 5 ensures that the product can be consumed with minimal oral manipulation while maintaining sufficient structural integrity to reduce aspiration risks, offering a nutritionally dense alternative to conventional starch-thickened purees. Accordingly, this study aimed to tailor the texture of *Nemipterus virgatus* surimi gel to meet the Level 5 criteria via ultrasound-assisted PG deamidation. This study examined the impact of PG deamidation treatment on the gel characteristics of surimi gels and employed ultrasound-assisted techniques to enhance the gel softening effect. This study examined the efficacy of the treatment on gel digestion through in vitro digestion simulation experiments. The mechanism of ultrasound-assisted enzyme deamidation treatment on the molecular conformation of myofibrillar proteins was further examined. The deamidation of PG softens surimi gels, demonstrating the viability of producing soft surimi gels and offering a novel approach for developing surimi products that ensure swallowing safety and nutritional efficacy.

## 2. Materials and Methods

### 2.1. Materials

Frozen surimi from *Nemipterus virgatus* were purchased from the Qingdao Tonbway Foodstuffs Co., Ltd. (Qingdao, China). Fillets of *Nemipterus virgatus* were purchased from the Jinzhou Haiying Aquatic Products Store (Jinzhou, China). Protein-glutaminase (PG) (500 U/g) were purchased from Kinry Food Ingredients Co., Ltd. (Shanghai, China). All other chemicals were at least of analytical grade.

### 2.2. Preparation of Surimi Gels

The surimi, frozen at −80 °C, was positioned in a semi-thawed condition. Process them in UMC 5 vacuum cutter-mixer (Stephan Machinery GmbH, Hameln, Germany) for three minutes. Surimi was incorporated with 1.5% (*w*/*w*) salt and 0.75% (*w*/*w*) compound phosphate and processed at atmospheric pressure for 3 min. Ice water was incorporated to achieve a moisture content of 85%, and the surimi paste was produced by chopping for 3 min. Upon adding the enzyme, it is combined with ice water. Subsequently, the surimi paste was encased in a 32 mm diameter casing and sealed at both ends. The surimi sausages were incubated at a low temperature of 10 °C for 2 h. The surimi gel was prepared using a two-stage water bath heating process at 4 °C for 30 min, followed by 90 °C for 20 min. Finally, all samples were stored at 4 °C for subsequent analysis. Notably, to ensure comparability across all treatment groups in the subsequent digestion and protein structure analyses, the initial weight of frozen surimi added to each batch was kept constant (all groups started with the same amount of raw surimi material, which contained approximately 15% protein). Therefore, despite the differences in final moisture content (78% vs. 85%), the total initial protein load was uniform, allowing direct comparisons of peptide and free amino acid concentrations without requiring additional normalization to protein content.

### 2.3. Extraction of Myofibrillar Protein (MP)

MP was extracted utilizing the method of Alvarez et al. [16]. with minor modifications. A quantity of back muscle from *Nemipterus virgatus* was weighed and ground using a meat grinder. Four times the volume of a phosphate buffer solution, with a pH of 7.5 and a concentration of 20 mmol/L, was added to the minced meat (S18-LA170, Joyoung Co., Ltd., Jinan, China). The mixture was homogenized by T18 brushless digital ultra-turrax (IKA-Werke GmbH and Co. KG, Staufen, Germany) at 5000 rpm for 90 s, subjected to three homogenization cycles. The samples were subsequently centrifuged using a centrifuge (Biofuge Stratos, Thermo Fisher Scientific, Waltham, MA, USA). The entire procedure was repeated twice. The precipitate was subjected to centrifugation after the addition of a fourfold volume of 0.1 mol/L NaCl solution, then homogenized three times at 7000 rpm for 1 min each, followed by further centrifugation. The precipitate was dissolved in six volumes of phosphate buffer with 0.1 mol/L NaCl and homogenized at 7000 rpm for 30 s. The mixture was filtered through four layers of gauze to isolate the precipitate, which was acquired as MP. The entire procedure was conducted at 4 °C. Centrifugation was executed at 7500 revolutions per minute for 15 min, also at 4 °C. The subsequent experiments on MP were conducted using a phosphate buffer with 0.6 mol/L NaCl and a pH of 7.5 at a concentration of 20 mmol/L.

### 2.4. Preparation of MP Gel

The MP concentration was modified to 80 mg/mL. PG was incorporated into MP at a concentration of 16 U/g protein. Consequently, two groups were subjected to sonication utilizing an ultrasound device (KQ-400KDE, Kunshan Ultrasonic Instruments Co., Ltd., Kunshan, China). The sonication duration was 31 min, the sonication power was 240 watts, and the cryogenic reaction duration was 59 min. The samples were positioned in gel vials and subjected to heating in a water bath (HH.S11-2, Shanghai Boxun Medical Biological Instrument Corp., Ltd., Shanghai, China) at 40 °C for 30 min and at 90 °C for 20 min to produce MP gels. The samples were refrigerated at 4 °C overnight.

### 2.5. Study on Gel Properties of Soft Surimi Gel

#### 2.5.1. Texture Profile Analysis (TPA)

The surimi gel samples were equilibrated to room temperature and subsequently divided into cylinders approximately 20 mm in height for the assessment of TPA utilizing a TA-XT-Plus texture analyzer (Stable Micro Systems Ltd, Surrey, UK) [17]. The probe model was P50, featuring a trigger force of 5 g. The pre-test speed, test speed, and post-test speed were uniformly 1 mm/s, accompanied by a compression percentage of 40%.

#### 2.5.2. Determination of Gel Strength

The gel strength was assessed utilizing a TA-XT-Plus texture analyzer [18]. The probe model was P/5S, the trigger force was 5 g, the downward pressure distance was 15 mm, and the pre-test, test, and post-test speeds were all 1 mm/s, with six replicates in each group. The gel strength of the sample was assessed as follows:

Gel strength (g mm) = Fracture force (g) × Fracture Distance (mm)(1)

#### 2.5.3. Whiteness Values

The luminance value (L*), red-green value (a*), and yellow-blue value (b*) of the gel were measured using a colorimeter (model CR-400, Konica Minolta Sensing, Inc., Tokyo, Japan). Whiteness values (W) was assessed as follows:

Whiteness values (W) = 100 − [(100 − L*)^2^ + a*^2^ + b*^2^]_1/2_ (2)

#### 2.5.4. IDDSI Test

The International Dysphagia Diet Standardization Initiative (IDDSI) established guidelines for modifying food texture based on various food types and comprehensive classification standards for consumable items, employing an 8-grade scale (0–7 grades) [19]. The testing methods for foods within the framework comprise the fork pressure test, spoon pressure test, and spoon tilt test.

#### 2.5.5. Determination of Degree of Deamidation

Determination of degree of deamidation was assessed utilizing an ammonia assay kit (Catalog No. MAK310, Sigma-Aldrich, St. Louis, MO, USA), adhering to the methodology of Fu et al. [11]. with minor modifications. The gel samples were combined with pure water in a 1:5 ratio and homogenized at 10,000 rpm for 90 s. The supernatant was centrifuged at 10,000× *g* for 10 min and subsequently diluted 50-fold with distilled water. The standard curve for ammonia concentration was constructed utilizing NH_4_Cl standards. Ten microliters of diluted sample were combined with ninety microliters of ammonia detection reagent and allowed to react for fifteen minutes in a light-protected environment. The fluorescence intensity was subsequently measured using a multimode microplate reader (Victor3, PerkinElmer, Inc., Waltham, MA, USA), and the ammonia concentration (N_1_) was determined. The excitation wavelength measured 360 nm, while the emission wavelength was recorded at 460 nm. The total protein-releasable ammonia concentration (N_2_) was ascertained by combining the homogenized gel solution in a 1:1 ratio with a 6 mol/L HCl solution and heating at 95 °C for 4 h. The extent of deamidation was assessed as follows:

Determination of degree of deamidation = (*N*_1_/*N*_2_) × 100% (3)

#### 2.5.6. Water Holding Capacity and Cooking Loss

The water holding capacity was assessed using the methodology of Lv et al. [20], while the cooking loss was evaluated according to the approach of Nie et al. [21], with minor modifications. The water holding capacity was assessed as follows:

water holding capacity (%) = *W*_2_/*W*_1_ × 100% (4)

where W_1_ and W_2_ are the weight of the gel before cooking (g) and the weight after cooking, respectively.

Cooking losses was assessed as follows:

cooking loss (%) = (*G*_1_ − *G*_2_)/*G*_1_ × 100% (5)

where G_1_ and G_2_ are the weights (g) of the original gel before and after centrifugation, respectively.

#### 2.5.7. Moisture Distribution and MRI Imaging Determination

The moisture distribution of surimi gels was assessed using the method of Zhang et al. [22], with minor modifications. The lateral relaxation time T_2_ of the gel samples was ascertained using low-field NMR (NMI20, Shanghai Electronic Technology Co., Ltd., Shanghai, China). Parameter settings: Spectral Width (SW) = 100 kHz; Receiver Gain (RG) = 15; Digital Gain (DG) = 3; Relaxation Delay (D1) = 3000 ms; Number of Scans (ns) = 8; Echo Time (TE) = 0.2 ms; Number of Echoes (NE) = 5000.

Proton density-weighted images of the gel samples were acquired using low-field NMR (NMI20, Shanghai Electronic Technology Co., Ltd., Shanghai, China). Parameter settings: Spectrometer Frequency (SF) = 21.14 MHz; 90° Pulse Width (P1) = 12.40 μs; 180° Pulse Width (P2) = 22.00 μs; 90° Pulse RF Amplitude (RFA1) = 1.3%; 180° Pulse RF Amplitude (RFA2) = 1.9%; Receiver Gain (RG) = 20.0 dB.

#### 2.5.8. Observation of Gel Microstructure

Microscopic analysis was conducted according to HE staining routine protocols, with a biological microscope (BHT-312, Olympus Co., Ltd., Tokyo, Japan) to observe extracellular space and fiber shrinking in tissue.

Scanning electron microscopy (S-4800, Hitachi Co., Ltd., Tokyo, Japan) was used to observe microstructure and morphology changes [23]. The samples were examined with a scanning electron microscope at an accelerating voltage of 3.0 kV and a magnification of 500×.

### 2.6. Response Surface Methodology

Experiments were performed examining ultrasound time, ultrasound power, and ultrasound low-temperature reaction time as individual variables. Hardness was selected as the sole response variable for RSM optimization, because it directly reflects the mechanical resistance that must be overcome during oral processing. According to the IDDSI framework, the transition from Level 6 to Level 5 is primarily governed by the ease of compression and mastication, with hardness being the most straightforward and objective textural parameter to quantify. Furthermore, preliminary correlation analysis indicated that hardness was strongly correlated with gel strength (R^2^ > 0.95), confirming its suitability as a representative parameter for optimizing gel softening targeted at dysphagia-oriented applications. The hardness served as the response variable for the identification of key factors in the single-factor experiment, as illustrated in Table S1.

### 2.7. Study on In Vitro Digestion Characteristics of Soft Surimi Gel

#### 2.7.1. In Vitro Gastrointestinal Digestion

In vitro static simulated digestion method, referencing Bai et al. [24], with minor modifications. Ten grams of surimi gel samples were minced and homogenized with ten milliliters of distilled water (10,000× *g*, 1 min), then combined with twenty milliliters of simulated gastric fluid. Simulated gastric fluid consisted of 0.32% (*w*/*v*) pepsin (Sigma-Aldrich, St. Louis, MO, USA, ≥250 U/mg) in 0.03 mol/L NaCl, adjusted to pH 2.0 with 1 mol/L HCl. The heterogeneous solution was titrated to pH 2.0 using 1 mol/L HCl or NaOH and subsequently agitated at 37 °C for 2 h. Subsequent to the reaction, the mixed solution was calibrated to a pH of 7.2 using a 0.5 mol/L NaOH solution. During the simulated intestinal digestion phase, 20 mL of the aforementioned gastric digestive juice was combined with 20 mL of simulated intestinal fluid and incubated in a 37 °C water bath for 2 h. Simulated intestinal fluid consisted of 1% (*w*/*v*) pancreatin (Sigma-Aldrich, 8 × USP) and 0.6% (*w*/*v*) bile salts in 0.05 mol/L KH_2_PO_4_ buffer, adjusted to pH 7.2 with 0.5 mol/L NaOH. Following the reaction, it was terminated by heating with boiling water at 95 °C for 5 min and subsequently centrifuged at 10,000× *g* for 15 min. The supernatant was harvested and preserved at 4 °C.

#### 2.7.2. Determination of Digestibility

The protein concentration C_2_ (mg/mL) in the supernatant and the total protein concentration C_1_ (mg/mL) in the sample were quantified using the biuret method [25]. The in vitro protein digestibility was assessed as follows:

Digestibility (%) = *C*_2_/*C*_1_ × 100 (6)

#### 2.7.3. Peptide Composition Determination

The relative molecular mass and distribution range of collagen peptide were determined using high-performance size exclusion chromatography. Following the method outlined by Teng et al. [26], the concentration of peptide composition was quantitatively assessed by liquid chromatography (LC-2050C 3D, Shimadzu Corporation, Kyoto, Japan). The chromatographic column measured 300 mm × 7.8 mm TSK gel G2000 SWXL (Tosoh Corporation, Tokyo, Japan), with a temperature of 30 °C. The detection wavelength was 220 nm, the injection volume was 10 μL, and the flow rate was 0.5 mL/min. The mobile phase consisted of acetonitrile, water, and trifluoroacetic acid in a ratio of 40:60:0.05.

#### 2.7.4. Determination of Free Amino Acids

The sample was uniformly mixed, and 0.2 g of the sample solution along with the internal standard solution was placed into a 10 mL sealed glass tube with a stopper. Subsequently, 200 μL of the sample solution, 1 mL of sodium carbonate buffer solution, and 200 μL of a 3% 2,4-dinitrochlorobenzene derivative acetonitrile solution were added. The reaction was conducted in a constant-temperature water bath at 95 °C for 150 min in darkness. Subsequent to cooling, 200 μL of 10% glacial acetic acid was introduced to neutralize the pH, and the supernatant was extracted via the organic membrane and analyzed using liquid chromatography (ARC 2489, Waters Corporation, Milford, MA, USA).

### 2.8. The Determination of MP Structure by Ultrasonic-PG Treatment

#### 2.8.1. Determination of Intermolecular Forces

The assessment of intermolecular interactions is founded on Wei’s method with slight modifications [27]. Two grams of gel samples were incorporated into 10 mL of solutions A, B, C, D, and E, respectively, and designated as A_F_, B_F_, C_F_, D_F_, and EF. The samples were homogenized at 10,000 rpm for 1 min and subsequently stored in a refrigerator at 4 °C for 1 h. The sample was then centrifuged at 8000× *g* for 15 min to eliminate the supernatant. Liquid A comprised 0.05 mol/L NaCl, liquid B contained 0.6 mol/L NaCl, liquid C consisted of 0.6 mol/L NaCl with 1.5 mol/L urea, liquid D included 0.6 mol/L NaCl with 8 mol/L urea, and liquid E was a composite solution of 0.6 mol/L NaCl with 8 mol/L urea and 0.6 mol/L β-mercaptoethanol.

The protein concentration in the supernatant was quantified using the Coomassie Blue method at 595 nm. 100 μL of AF liquid supernatant was combined with 5 mL of Coomassie Blue solution, and the resultant measurement was documented as S_A_. 10 μL of B_F_/C_F_/D_F_ supernatant was combined with 90 μL of B/C/D solution and 5 mL of Coomassie Blue solution, with the measurement recorded as S_B_/S_C_/S_D_. 5 μL of EF liquid supernatant was combined with 95 μL of E liquid and 5 mL of Khomas Brilliant Blue solution, and the measurement was documented as SE. Assessment of the magnitude of each chemical forcing:

(7) $$\left\{\begin{array}{c} Ionic\;bonds\;=\;S_{B}-S_{A}\\ Hydrogen\;bond\;=\;S_{C}-S_{B}\\ Hydrophobic\;interactions\;=S_{D}-S_{C}\\ Disulfide\;bonds\;=S_{E}-S_{D} \end{array}\right.$$

#### 2.8.2. Determination of Secondary Structure

The gel samples were sectioned into thin slices and positioned on slides. The Raman spectra of the gels within the wavelength range of 400–2000 cm^−1^ were acquired using Horiba LabRAM HR Evolution spectrometer (Horiba, Kyoto, Japan) with an excitation wavelength of 532.

#### 2.8.3. Determination of Tertiary Structure

3D-EEM spectroscopy were obtained with minor modifications based on the methodology of Li et al. [28]. The samples were diluted to 0.5 mg/mL with phosphate buffer and analyzed using an F-4700 fluorescence spectrophotometer (HITACHI, Tokyo, Japan). The 3D-EEM spectroscopy were acquired within the excitation range of 200 to 340 nm and emission range of 275 to 550 nm, utilizing a slit width of 5 nm and a scanning velocity of 12,000 nm/min.

A minor alteration of intrinsic fluorescence spectroscopy was implemented with regard to Ren et al. [29]. The samples were diluted to 0.1 mg/mL with a hydrogen phosphate buffer solution and analyzed using an F-4700 fluorescence spectrophotometer (HITACHI, Tokyo, Japan). The excitation wavelength was 280 nm, the excitation slit width was 5 nm, the scanning rate was 200 nm/min, and the emission wavelength range was 300–450 nm.

### 2.9. Statistical Analysis

Each experiment was conducted thrice, and the variance was analyzed. The results were presented as mean ± standard deviation. Charts were generated utilizing Origin 2025 (OriginLab Corporation, Northampton, MA, USA). One-Way Analysis of Variance (ANOVA) and Tukey’s test were conducted utilizing SPSS (version 20.0, Chicago, NY, USA). The threshold for statistical significance was established at *p* < 0.05.

## 3. Results and Discussion

### 3.1. Analysis of Gel Properties of Soft Surimi Gel

#### 3.1.1. Analysis of Gel Strength and Textural Characteristics

Gel strength and textural characteristics are the primary metrics for assessing the quality of surimi gels. Table 1 indicates that gel hardness, elasticity, cohesion, and chewiness were maximized in the absence of PG, with a hardness measurement of 607.40 ± 10.26 g. As the quantity of enzyme added increased, the gel’s hardness, elasticity, cohesion, and chewiness diminished, with the rate of decrease stabilizing at 0.2% of the added amount, attributable to alterations in the network structure of the surimi gel resulting from the deamidation treatment. The trend of gel strength correlated with the trend of hardness.

**Figure 1:**
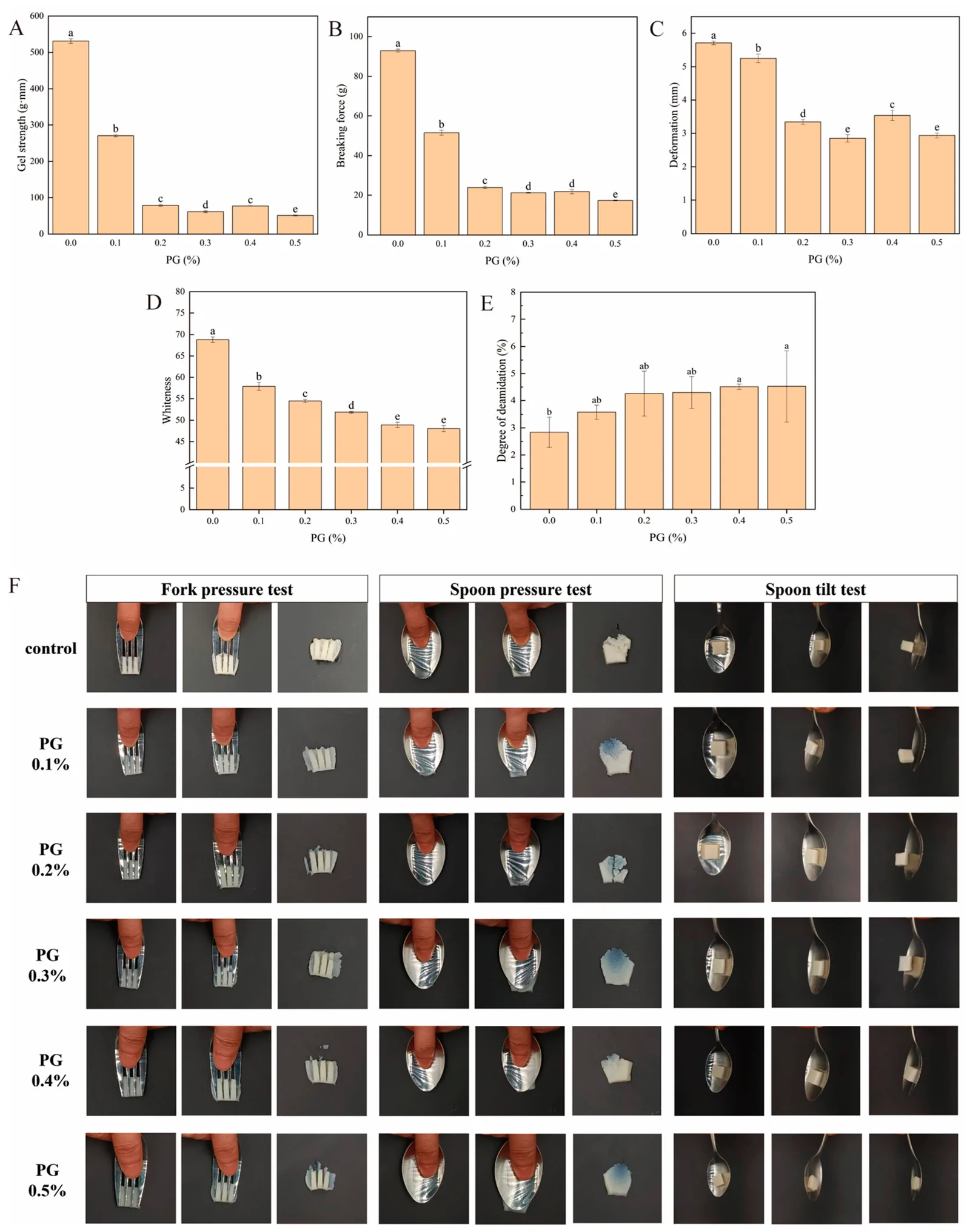
The influence of PG incorporation on the gel strength (**A**), breaking force (**B**), breaking distance (**C**), whiteness (**D**), degree of deamidation (**E**), and experimental results of IDDSI (**F**) of surimi gel with different PG additions. Different PG additions were designated as 0%, 0.1%, 0.2%, 0.3%, 0.4%, and 0.5%, the same as below. Different letters (a–e) indicate significant differences among different treatments (*p* < 0.05).

Figure 1A illustrates that the addition of 0.1% PG markedly reduced the gel strength of fish guts from 530.79 g mm to 270.21 g mm (*p* < 0.05). With the addition of 0.2% PG, the gel strength diminished to 78.61 g mm. Despite the ongoing increase in addition, the gel strength remained relatively unchanged. The breaking force indicates the damage resistance of the gel and is positively correlated with its strength. The breaking force diminished from 92.97 g to 51.46 g with the incremental addition of enzyme, indicating that enzyme supplementation reduces the force necessary for the breakdown of surimi during oral processing. The reduction in gel strength was ascribed to modifications in protein conformation resulting from the alteration of the cross-linking mechanism of the protein network due to PG deamidation. PG deamidation transforms glutamine (Gln) into glutamic acid (Glu), introduces negatively charged moieties, enhances electrostatic repulsive forces, and disrupts hydrophobic interactions among protein molecules, thereby compromising the structural integrity of the gel network [30].

Consequently, utilizing PG to deamidify surimi gels for the creation of soft gels intended for dysphagia food development is viable.

#### 3.1.2. Analysis of Whiteness Results

The color of surimi is affected by the color characteristics of surimi and the characteristics of additives [31]. With the increased enzyme addition, the whiteness value showed a significant decreasing trend (*p* < 0.05), as shown in Figure 1D. This phenomenon is attributed to the fact that PG itself is brown, which reduces the whiteness of surimi gel. The change of gel structure caused by PG caused the increase of light absorption on the gel surface, the decrease of brightness, the decrease of b*, and the color further shifted to blue. As more PG was added, the rate of change in whiteness decreased. Compared with other surimi, the whiteness value of *Nemipterus virgatus* surimi itself is low, and the whiteness value of the control group is 68.81. This difference is because the *Nemipterus virgatus* is a red meat fish with more myoglobin content.

#### 3.1.3. Analysis of Deamidation Degree Results

This study investigated the softening of surimi gel through PG deamidation, necessitating the assessment of the correlation between the quantity of PG incorporated in the deamidation reaction and the enzyme’s efficacy. Figure 1E illustrates that the extent of deamidation catalyzed by PG escalated with the increment of enzyme concentration; however, the rate of deamidation’s increase diminished when the enzyme concentration reached 0.2%. The introduction of a minimal quantity of enzyme can effectively and selectively transform Gln residues into Glu residues rapidly, resulting in alterations to the gel network structure. The alteration of the gel network impacts the substrate contact site, resulting in no substantial enhancement of enzyme efficiency, although the deamidation reaction persists. The overall low level of deamidation may be associated with the distinct structure of protein substrates [11].

#### 3.1.4. Experimental Results of IDDSI

The classification of surimi soft gels was further assessed through the fork and spoon experiment referenced by IDDSI. According to IDDSI framework, Level 5 (Minced & Moist) requires food particles not exceeding 4 mm in size, no dripping liquids, and the ability to be easily mashed with a fork. Our gels met these criteria through fork pressure test (easy indentation without fingernail blanching), spoon pressure test (easy compression without return to original shape), and spoon tilt test (sample held shape and fell off upon tilting) [19]. Figure 1F illustrates that both the control group and the addition of 0.1% PG can be categorized as IDDSI Level 6 (Soft & Bite-Sized). The nails turn partially white when pressed by the fork. In the spoon pressure test, the food did not revert to its original form post-compression. In the spoon tilt test, the sample retains its original form, and all the food will fall when the spoon is inclined downward. In the 0.2% PG group, the fork pressure test indicated reduced finger pressure, enhanced gel penetration by the fork, and relatively facile separation. The fingertips did not turn white upon the application of pressure. In the spoon pressure test, the spoon can easily compress the food without returning it to its original form. In the spoon tilt test, the gel sample retains its original shape and subsequently falls when the spoon is inclined and gently shaken. Consequently, it is categorized as IDDSI Level 5 (Minced & Moist). The 0.3% PG group was similar to the above results. During the spoon tilt test, the gel sample retained its original form and entirely dislodged when the spoon was inclined and gently agitated, although an increase in gel viscosity was perceptible. The group remains classified as Level 5. The 0.4% and 0.5% PG groups demonstrate ease in completing the fork pressure test and spoon pressure test; however, the spoon tilt test fails to slide down smoothly, indicating that these two groups cannot be categorized into Level 5 classification. The heightened viscosity poses a risk to patients during consumption.

#### 3.1.5. Analysis of Water Behavior in Surimi Gels

**Figure 2:**
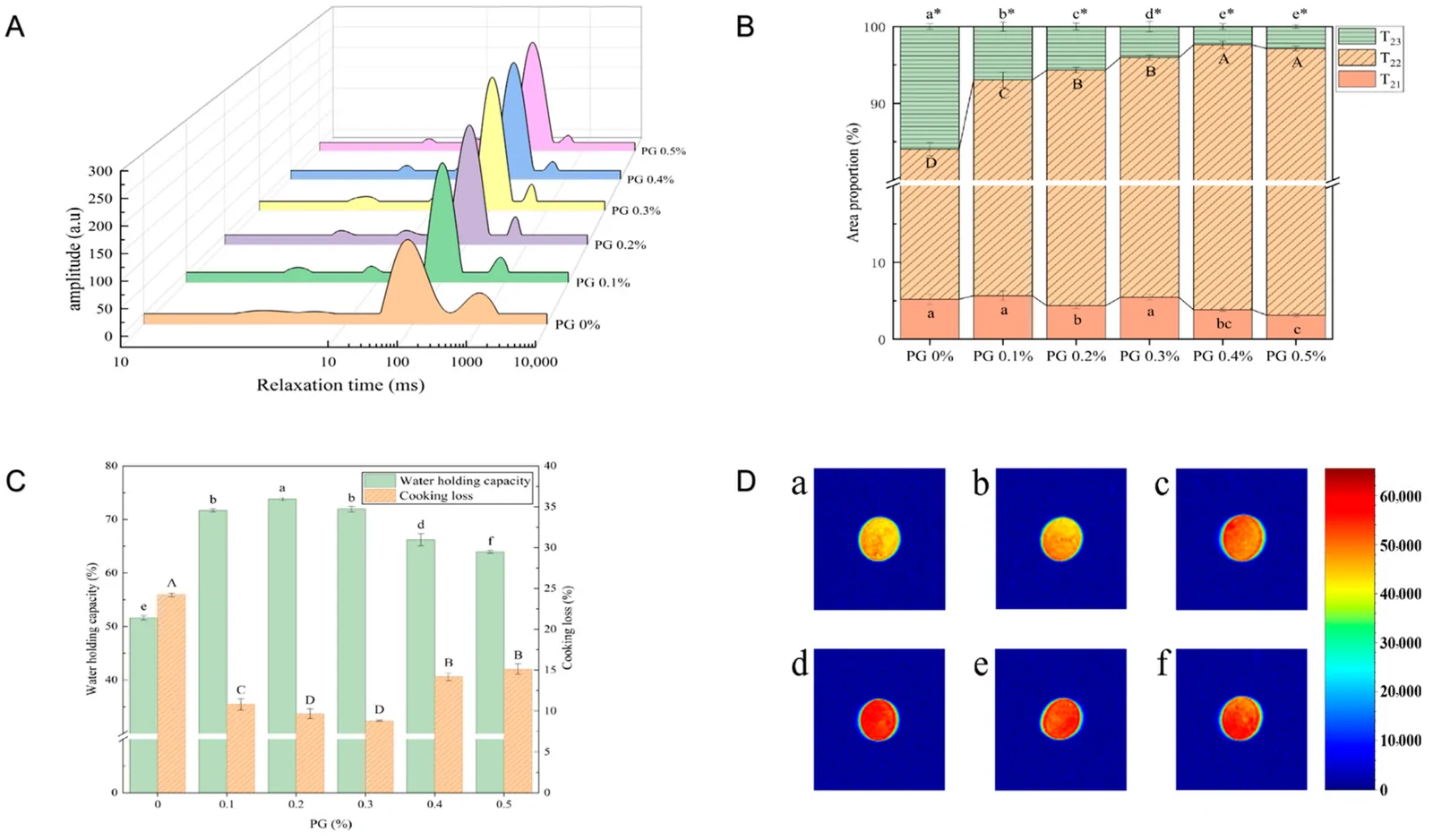
The impact of PG incorporation on the whiteness of surimi gel: water distribution (**A**,**B**), water holding capacity (**C**), cooking loss (**C**) and proton density-weighted images (**D**). (**D**) (**a**–**f**) represent PG additions of 0%, 0.1%, 0.2%, 0.3%, 0.4%, and 0.5%, respectively. In Figures (**B**,**C**), Different letters (a–f, A–D) indicate significant differences among different treatments (*p* < 0.05).Different superscript letters with asterisks (a*, b*, c*, d*, e*) indicate significant differences (*p* < 0.05) in the total water proportion among treatments.

Figure 2C illustrates that the cooking loss rate exhibited an inverse relationship with the water holding capacity. The augmentation of enzyme addition markedly improved the gel’s water holding capacity and substantially decreased the cooking loss rate. At an additional amount of 0.2%, the gel’s water holding capacity attained a maximum value of 73.81%, representing a 42.96% increase compared to the control group (*p* < 0.05). When the additive quantity exceeded 0.2%, the water holding capacity of the surimi gel diminished. At an addition level of 0.3% PG, the cooking loss of surimi gel attained a minimum value of 8.81%, representing a 63.63% reduction compared to the control group (*p* < 0.05). The rationale for the aforementioned modifications is that PG deamidation treatment enhances the negative charge density on the protein surface, thereby attracting more water molecules via electrostatic interactions. The treatment of silver carp MP with PG demonstrated that deamidation could enhance the water holding capacity of the protein [30]. The significant changes in cooking loss and water holding capacity were firstly related to the high initial moisture content during the preparation of surimi gels. In the experiment, the control group exhibited a layer of water on its surface, which was rough. Upon the addition of PG, the gel’s surface exhibited no moisture and appeared relatively even.

The transverse relaxation time (T_2_) in low-field NMR is employed to examine the fluidity and distribution of water [22]. Figure 2A illustrates that each sample group exhibited 3–4 distinct peaks, corresponding to varying relaxation time values (T_2_). Figure 2B demonstrates that the control group showed the greatest peak area associated with the relaxation time (T_23_) within the range of 100–1000 ms. The current free water content is elevated, and this portion of water molecules is nearly unbound by intermolecular forces. The incorporation of the enzyme elevated the percentage of immobilized water in the gel network from 78.85% to 84.40%, while the percentage of free water diminished from 15.95% to 6.94%, demonstrating that deamidation treatment restricted the fluidity of water within the surimi gel network (*p* < 0.05). This decrease may be associated with the substantial quantity of carboxylate ions generated by the deamidation treatment, which establishes robust hydrogen bonds with water and elevates the fraction of less mobile water through chemical interaction. The rise in the proportion of less mobile Water elucidated the enhancement of water holding capacity and the reduction of cooking loss in surimi gel.

Generally, an increase in moisture content in the sample correlates with a redder hue in proton density-weighted images, while a decrease correlates with a bluer hue [32]. Figure 2D illustrates that as the quantity of enzyme addition increases, the surimi gel sample transitions from yellow to red. At a PG addition of 0.3%, the samples exhibited the darkest NMR imaging color, signifying that this group experienced the least water loss and reduced water mobility during preparation. The formation of thermally induced gel resulted in the conversion of substantial amounts of water into less mobile water retained within the gel network. The hue of the 0.4% and 0.5% addition groups became marginally lighter, potentially attributable to the transformation of bound water into less mobile water within the water distribution.

#### 3.1.6. Impact of PG on the Microstructure of Surimi Gel

**Figure 3:**
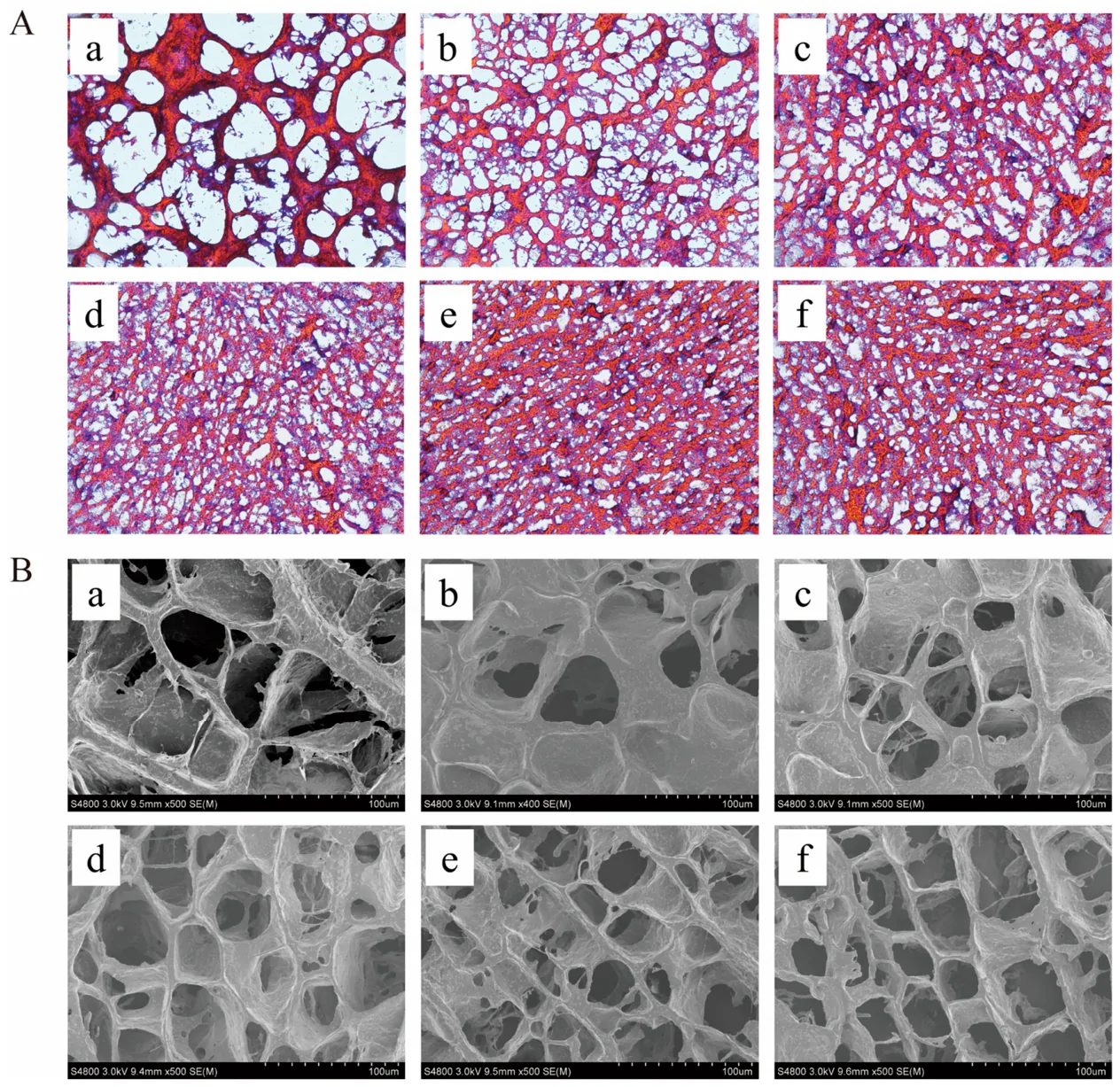
Impact of PG on the microstructure of surimi gel: HE staining (**A**) and scanning electron microscopy (SEM, (**B**)). For HE staining (**A**), red-pink areas correspond to the protein network, and white areas represent gel pores. Panels (**a**–**f**) represent PG additions of 0%, 0.1%, 0.2%, 0.3%, 0.4%, and 0.5%, respectively.

HE staining can indicate the microstructural alterations of surimi gel [33]. Figure 3 illustrates that the network structure of surimi gel samples with the addition of PG exhibited a markedly dense network configuration. In comparison to the 0% PG addition group, an increase in enzyme addition resulted in a denser HE staining area, a reduction in the thickness of the network’s filamentous structure, and a decrease in network pore size (Figure 3A). The composite gel’s microstructure was examined using scanning electron microscopy, providing a clearer representation of the alterations in the gel network structure (Figure 3B). In comparison to the control group, the pores of the gel fiber network with 0.1% enzyme addition diminished in size. As the quantity of enzyme added increased, the gel network became denser, the filamentous structure of the network thinned, and the square network structure grew increasingly compact. The results confirm that the change in gel network density does not necessarily correlate with the gel strength. The PG deamidation treatment converts Gln to Glu, thereby augmenting the protein’s negative charge, disrupting the original gel network structure, and facilitating recombination. The novel gel network is reconstituted by weak chemical interactions, such as hydrogen bonds, which may result in structural instability and diminished gel strength. Research indicates that deamidation treatment of chicken MP gels alters the protein’s secondary structure, resulting in the conversion of β-sheet expansion to α-helix [34]. The density of the network may correlate with the water-holding capacity of surimi gel. The incorporation of a minimal quantity of enzyme enhances the negative charge density on the protein’s surface, and the compact network structure facilitates improved retention of water within the gel matrix, reducing its loss.

### 3.2. Results of Optimization for Processing Parameters in Ultrasonic-Assisted PG Deamidation

Ultrasound treatment can induce the denaturation of protein structures, thereby revealing additional active sites for binding with exogenous additives [35]. As shown in Figure S1A–C and Tables S2–S4, the gel strength, hardness, and cooking loss were minimized at an ultrasonic duration of 30 min, as established by the single-factor experiment. At an ultrasonic power of 240 W, the gel strength, hardness, and cooking loss were minimized, while the water-holding capacity was markedly improved. At a low-temperature reaction time of 60 min, the gel strength, hardness, and cooking loss were minimal, while the water holding capacity was markedly improved and subsequently exhibited little variation. The response surface analysis (Tables S5 and S6 and Figure S2) indicated that the primary and secondary factors influencing the softening degree of surimi gel were ultrasonic power, ultrasonic time, and enzyme reaction time. The optimal conditions for ultrasonic-assisted deamidation of surimi gel, in conjunction with the experimental parameters, were as follows: Ultrasonic duration was 31 min; ultrasonic power was 240 W; the low-temperature reaction duration was 59 min.

### 3.3. Effects of Ultrasound and Enzyme Treatment on the Digestion Characteristics of Surimi Gel

#### 3.3.1. Analysis of Digestibility Change

**Figure 4:**
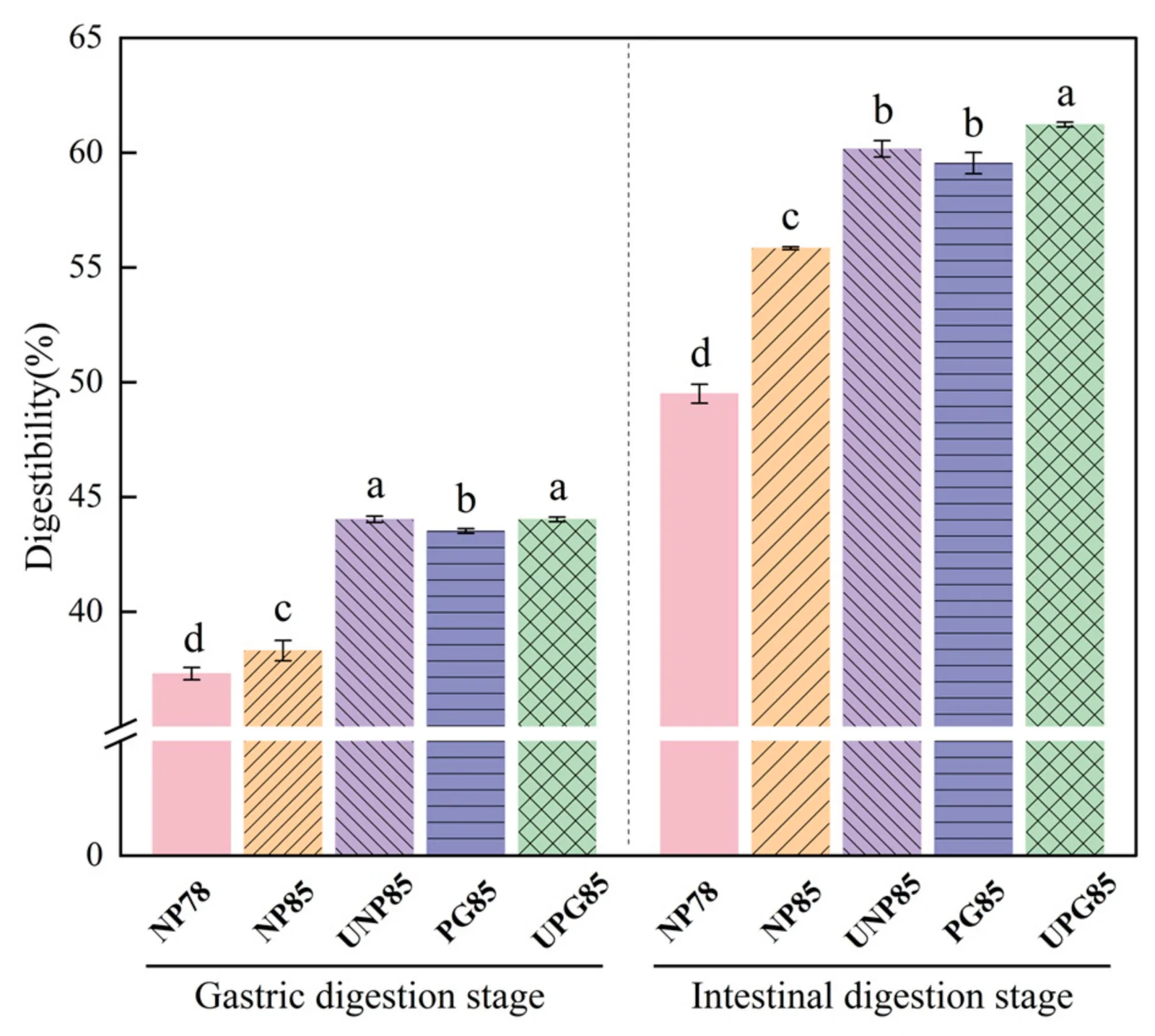
Effects of ultrasound and PG treatment on in vitro digestibility of surimi gel. Different letters (a–d) indicate significant differences among different treatments (*p* < 0.05). NP78 denotes a gel with 78% moisture content, while NP85 and PG85 signify 85% moisture content in the absence and presence of PG treatment, respectively. The surimi gels from the NP85 and PG85 groups underwent ultrasonic treatment prior to the low-temperature reaction, designated as UNP85 and UPG85, respectively.

Figure 4 illustrates that the trend of in vitro digestibility of surimi gel was analogous between the two digestion stages. The surimi gels from the NP85 and PG85 groups underwent ultrasonic treatment prior to the low-temperature reaction, designated as UNP85 and UPG85, respectively The NP78 group exhibited reduced water content, and the gel network structure was more tightly integrated during the heat-induced formation of surimi gel cross-linking. Consequently, the gel binding sites were less accessible, impeding the interaction between digestive enzymes and substrates, resulting in diminished digestibility. Ultrasonic treatment significantly enhanced the digestibility of surimi gel protein compared to the NP85 and UNP85 treatment groups. The instability of the gel network structure in high-moisture gel is due to ultrasound altering its configuration. During the gastric digestion phase, the gel network structure was disrupted by the influence of a highly acidic environment and pepsin. The ultrasound-induced cavitation effect enhanced the unfolding of the gel structure, increasing the exposure of contact sites, facilitating the binding of proteins and digestive enzymes, and improving gel digestibility. Relevant studies indicate that ultrasound-assisted low-salt air-dried fish markedly enhanced the NaCl transfer rate, augmented tenderness, and further improved water retention and in vitro digestibility [36]. The incorporation of PG enhanced protein digestibility, attributable to the deamidation treatment that facilitated the expansion of the gel’s network structure without inducing crosslinking under thermal conditions, thereby exposing a significant number of amino acid residues on the gel surface. Therefore, the in vitro digestibility of the soft gel prepared by ultrasound-assisted PG deamidation treatment was improved.

#### 3.3.2. Analysis of Peptide Distribution Change

Table 2 indicates that the molecular weight of the final digestion product in the NP78 group was 7.03%, surpassing that of the other groups. The distribution of small molecular weight peptides was inferior to that of other groups due to the enhanced stability of the low-moisture gel structure and insufficient substrate decomposition by digestive enzymes. The concentration of small molecules in the UNP85 group exceeded that in the NP85 group, and ultrasonic treatment enhanced the levels of small molecular weight peptides in gel digestion products. The cavitation effect induced by ultrasonic treatment facilitates the expansion of the gel structure and enhances crosslinking during the heating process. Whether or not the enzyme was introduced, the ultrasonic treatment of the high-moisture surimi gel would enhance the distribution of the final product of surimi gel digestion within the peptide segment under 1 kDa. In comparison to the NP85 and PG85 groups, the incorporation of the enzyme also elevated the concentration of small molecular weight peptides. The concentration of peptides with molecular weights below 500 Da was elevated, while the concentration of peptides exceeding 5 kDa was diminished, and the concentration of peptides ranging from 1 to 5 kDa was augmented. Research indicates a reduction in the concentration of high molecular weight peptides in the digestion products of deamidated proteins [37]. In comparison to the PG85 group, the concentration of small molecular peptides in the UPG85 group increased further, attributable to ultrasound facilitating the unfolding of the gel structure and enhancing the deamidation of the PG.

#### 3.3.3. Analysis of Free Amino Acid Content Changes

Impact of ultrasound and enzymatic treatment on the concentration of free amino acids in the in vitro digestive fluid of surimi gel Table 3 indicates the detection of 11 types of free amino acids, including the essential amino acids valine, methionine, leucine, phenylalanine, and lysine. In comparing the total amino acid content of NP78, NP85, and UNP85, the NP78 group exhibited a low-moisture gel characterized by a high concentration of gel protein of the same quality. The total concentration of free amino acids and essential amino acids generated during the hydrolysis process was substantial; however, the actual degree of digestion was low. The proportion of essential amino acids is low, although the digestibility of the high-moisture group is enhanced during digestion; however, the overall amino acid content and essential amino acid content show minimal variation. In the PG85 group, the alteration of protein structure and the augmentation of small molecular peptides resulting from deamidation led to the production of a greater quantity of small molecular peptides and free amino acids during digestion. The concentration of essential amino acids rose, and their proportion exceeded that of other groups. Consequently, ultrasound-assisted enzyme treatment of surimi gel promotes the production of free amino acids and essential amino acids, thereby suggesting enhanced protein digestibility in vitro of surimi gel.

### 3.4. Changes of MP Structure Subjected to Ultrasound and PG Treatment

#### 3.4.1. Analysis of Intermolecular Forces Change

**Figure 5:**
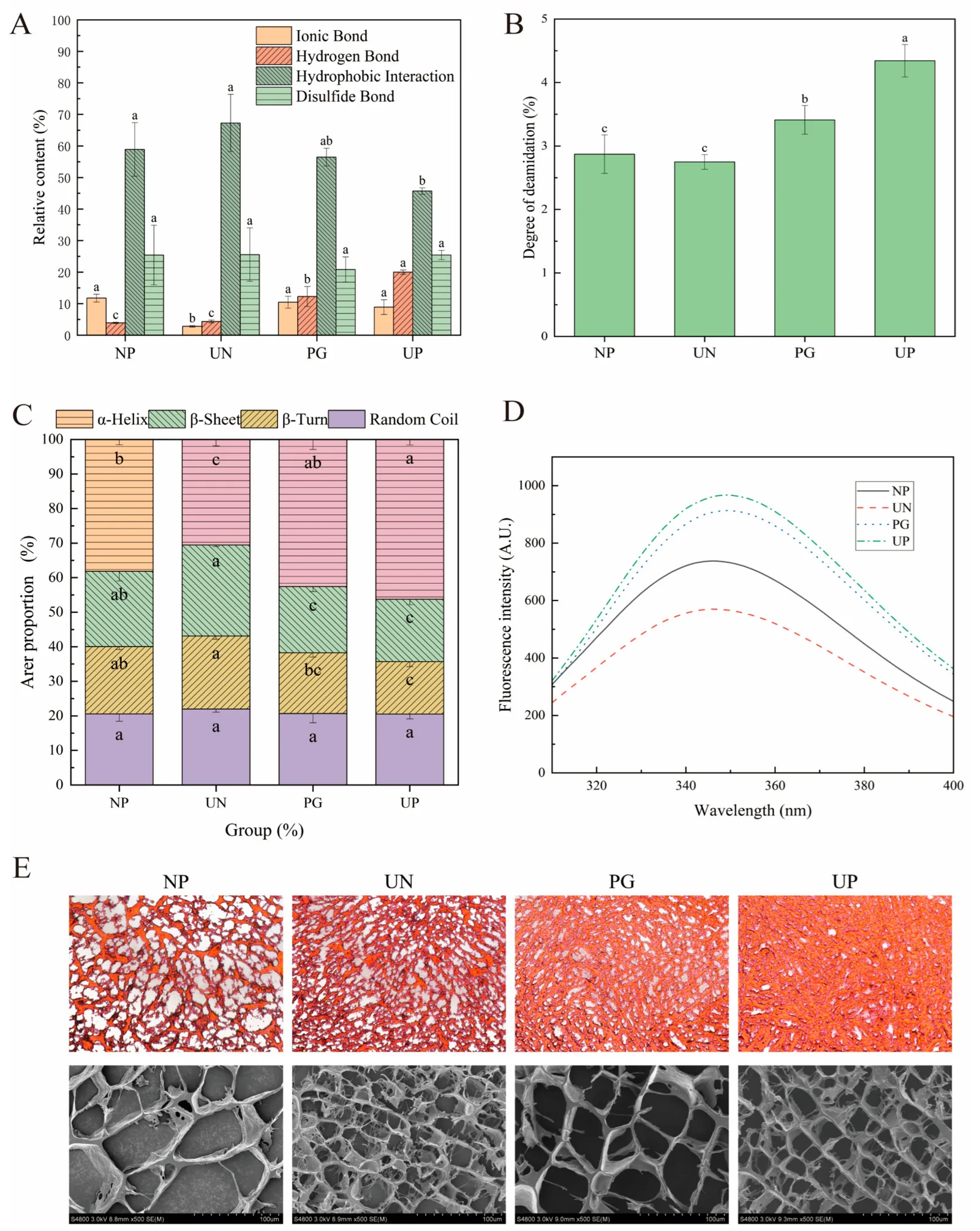
The changes in the intermolecular forces (**A**), deamidation degree (**B**), secondary structure (**C**), intrinsic fluorescence spectroscopy results (**D**) and microstructure (**E**) of myofibrillar protein gel following ultrasonic and enzymatic treatment.In panel E, the upper micrographs correspond to HE staining, where reddish-orange areas represent the protein network and white areas represent gel pores; the lower images are scanning electron micrographs. NP is the untreated control group; PG represents the protein-glutaminase treatment group. UN denotes the group subjected to ultrasound alone, and UP represents the combined ultrasound and PG treatment group. The same group nomenclature applies throughout the manuscript. Different letters (a–c) indicate significant differences among different treatments (*p* < 0.05).

The properties of gels are intricately connected to chemical forces, and gel formation typically occurs through the interaction of unexpanded MP via these chemical links [38]. Hydrophobic interactions constitute the fundamental framework for the formation of a protein gel network. Ultrasonic treatment was observed to augment the extractable fraction associated with hydrophobic interactions (Figure 5A). Following protein denaturation, the internal hydrophobic residues, such as phenylalanine and leucine, become exposed, prompting molecular aggregation through hydrophobic interactions to establish a gel network. The deamidation treatment appeared to diminish the hydrophobic interaction-related fraction while increasing the hydrogen bond-related extractable fraction. This shift in the balance of intermolecular forces may partially account for the reduction in gel strength and hardness, as weaker hydrogen bonds may provide less structural reinforcement than hydrophobic interactions in the gel network. Related studies have demonstrated that the application of PG to alter soy protein, glutelin, and gliadin transitions the primary chemical interactions among protein molecules from hydrophobic interactions or covalent bonds (Schiff base formation) to weakened van der Waals forces or hydrogen bond associations [39].

#### 3.4.2. Analysis of Deamidation Degree Change

Figure 5B illustrates that the impact of ultrasonic treatment on the extent of protein deamidation was not significant (*p* > 0.05). This maybe attributed to the ultrasonic cavitation effect, which facilitated the unfolding of the protein conformation, while the ensuing heat-deamidation treatment in the PG treatment group effectively and selectively transformed Gln residues into Glu residues, thereby enhancing the extent of protein deamidation. The degree of deamidation in the ultrasound-assisted enzyme treatment group was enhanced, attributable to alterations in the gel network, which exposed Gln residues and resulted in an increased level of protein deamidation.

#### 3.4.3. Analysis of Secondary Structure Change

Figure 5C illustrates that the α-helix of the UN group diminished from 38.17% to 30.57%, likely attributable to the disruption of hydrogen bonds caused by ultrasonic treatment, which led to the degradation of the α-helix structure. The increase in β-sheet content from 21.77% to 26.33% suggests enhanced protein-protein interactions, possibly due to the exposure of hydrophobic regions following ultrasonic treatment, which further enhanced protein-protein interactions, paralleling the findings of Li et al. [40]. The deamidation treatment elevated the α-helix structure proportion from 38.17% (NP) to 42.59%, while ultrasound-assisted treatment further augmented it to 46.27%. Concurrently, the β-sheet structure proportion diminished from 21.77% to 19.18%. One plausible interpretation is that the conversion of glutamine to glutamic acid residues through deamidation increases the net negative charge and electrostatic repulsion, which may disturb the inter-chain hydrogen bonding pattern and facilitate the unfolding of β-sheet structures, consistent with previous observations. However, direct evidence at the residue level would require additional high-resolution structural analyses [41]. During ultrasound-assisted treatment, increased enzyme contact sites were revealed following the unfolding of the protein structure, leading to deamidation, which inhibited the disordered cross-linking of the protein during the subsequent heat-induced gelation process. The enhancement of hydrogen bonds in the assessment of chemical forces should be closely associated with alterations in α-helix structure. The β-sheet structure is stable, whereas the α-helix is a ‘ring’ structure that is comparatively flexible and more pliable [42]. This conclusion elucidates the reduction in protein gel hardness and gel strength.

#### 3.4.4. Analysis of Tertiary Structure Change

**Figure 6:**
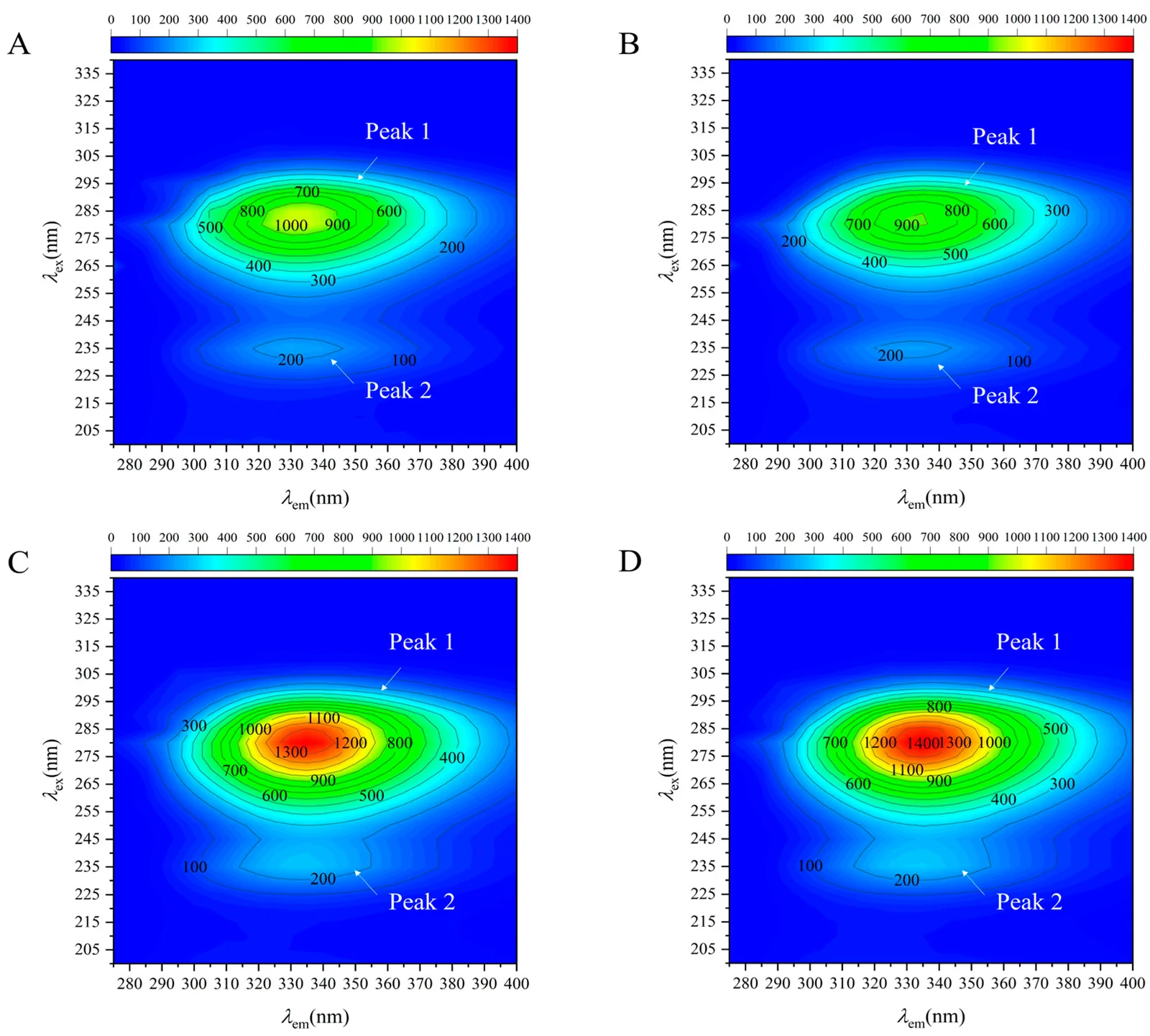
Effects of ultrasound and enzyme treatment on the 3D-EEM spectroscopy results of myofibrillar protein gel. (**A**–**D**) represent the treatment group control group, noted as NP, UN, PG, and UP, respectively.

Phenylalanine, tyrosine, and tryptophan are three essential amino acids in organisms and serve as crucial constituents of proteins. Peak 1 primarily pertains to the spectral characteristics of tyrosine and tryptophan residues, while Peak 2 predominantly relates to alterations in the polypeptide chain structure [28]. Compared to Figure 6A, Peak 1 in Figure 6B showed a diminished peak area and fluorescence intensity, indicating a reduction in the fluorescence intensity of tyrosine and tryptophan. Figure 5D shows a similar result, indicating that ultrasonic treatment results in a reduction of tryptophan fluorescence intensity. This decline is attributed to the protein structural unfolding induced by the cavitation and mechanical effects of ultrasound, leading to the fluorescence quenching of tryptophan and tyrosine residues. The enzymatic treatment does not induce fluorescence quenching. The deamidation treatment induces a modification in the protein structure while safeguarding the tryptophan and tyrosine residues from degradation. As shown in Figure 6C, the peak area and fluorescence intensity of Peak 1 were markedly greater than those depicted in Figure 6A, whereas the peak area and fluorescence intensity in UP group exceeded those in PG. This improvement is due to the fact that ultrasonic treatment enhances the deamidation of PG under protein conditions. The fluorescence data suggest that ultrasound treatment tends to induce quenching of tryptophan and tyrosine residues, whereas ultrasound-assisted deamidation treatment appears to protect these residues from quenching and may alter the hydrophobic microenvironment. A plausible explanation is that deamidation introduces charged carboxyl groups, thereby increasing the local polarity around tryptophan residues. Nevertheless, these fluorescence-based interpretations should be regarded as indicative of conformational changes rather than definitive proof of specific molecular events, given the possible confounding effects of scattering and aggregation. The protein structure’s unfolding is accompanied by deamidation, exposing tryptophan to a hydrophilic environment. The establishment of a hydrophilic environment may be attributed to the alteration of charge induced by deamidation treatment, which also accounts for the reduction in hydrophobic interactions.

#### 3.4.5. Analysis of Microstructure Change

HE staining revealed that ultrasonic pretreatment compromised the structure of the protein gel network, while heat-induced aggregation of numerous protein molecules and increased interactions facilitated the formation of a denser three-dimensional network, as shown in Figure 5E. Scanning electron microscopy reveals filamentous cross-linking within the dense protein network structures. The mechanical and cavitation effects induced by ultrasonic processing diminish particle size while enhancing solubility. The overlapping interactions among protein molecules in the aerogel during ultrasonic vibration facilitate protein aggregation [43]. Ultrasound treatment will dismantle the protein structure and reform smaller sub-aggregates [44]. The enzymatic deamidation reaction exhibited a similar trend to the ultrasonic treatment. The network structure became more compact, yet the arrangement was more orderly, attributable to alterations in chemical forces. Ultrasound-assisted enzymatic treatment demonstrated that the pores in the fundamental gel matrix of enzymatic treatment were systematically enlarged. The enzyme treatment will enhance the MP network structure but will not facilitate crosslinking to generate a disordered filamentous structure during the heat-induced gelation process. The aforementioned factors may contribute to the reduction in hardness and gel strength. The disparity between the outcomes of HE staining and scanning electron microscopy may stem from the enhancement of water holding capacity within the protein network due to PG treatment.

## 4. Conclusions

In conclusion, PG deamidation treatment markedly diminished the gel strength and hardness of *Nemipterus virgatus* surimi (*p* < 0.05). Upon the addition of PG at 0.2%, the hardness and gel strength of surimi gel diminished from 607.40 g and 530.79 g mm to 244.60 g and 78.61 g mm, respectively, in comparison to the control group, and the texture of the surimi gel meeting the IDDSI Level 5 classification criteria, which serves as an initial indication for potential suitability in dysphagia diets. It should be noted that IDDSI framework testing provides preliminary texture classification rather than clinical swallowing safety validation. Future studies incorporating video fluoroscopic swallowing study (VFSS) or fiberoptic endoscopic evaluation of swallowing (FEES) are needed to confirm swallowing safety. The PG deamidation treatment resulted in the transformation of free water into water that was not readily flowable within the surimi gel. The water retention capacity of surimi gel was markedly enhanced (*p* < 0.05). Microstructural analysis indicated that the deamidation treatment impeded the cross-linking of surimi gels during the heat-induced gelation process, leading to the development of a uniform and dense mesh structure. The low-temperature deamidation reaction was facilitated by ultrasonic treatment to enhance the pliability of the surimi gel under the tested PG concentration, and to further investigate the impact of ultrasound-assisted enzyme deamidation on the conformation of myofibrillar protein. It should be noted, however, that while ultrasound appeared to improve enzyme accessibility and deamidation efficiency, the present experimental design did not systematically optimize PG concentration in combination with ultrasound. Therefore, the potential for reducing enzyme dosage through ultrasound assistance remains to be demonstrated in future dose-response optimization studies. Ultrasound-assisted deamidation of surimi gels produces an increased quantity of small molecular peptides and free amino acids, suggesting enhanced protein digestibility in vitro of surimi gels, and may facilitate digestion based on in vitro simulation results and absorption. It should be noted that in vitro digestion results do not directly translate to in vivo absorption or nutritional status, which require clinical studies for confirmation. In comparison to the control group, the ultrasonic treatment group markedly enhanced the hydrophobic interaction of MP, whereas the ultrasonic-assisted enzyme deamidation treatment diminished the hydrophobic interaction, augmented the hydrogen bond, elevated the α-helix structure to 46.27%, and reduced the β-sheet structure to 19.18%, demonstrating a significant difference from the control group (*p* < 0.05). The ultrasound-assisted deamidation treatment altered the polarity of the tryptophan residue microenvironment, disrupting the hydrophobic effect and consequently impeding the further folding of the unfolded protein structure during heat-induced gel formation. The establishment of additional weak interactions causes the unfolded protein network to exhibit a uniform square pore configuration. This study shows that ultrasound-assisted deamidation technology can soften surimi products to satisfy dysphagia food requirements while preserving their nutritional value as a high-quality protein source, offering a novel option for dysphagia patients. From a practical perspective, the 31-min ultrasound treatment and 59-min enzymatic reaction represent moderate processing times that could be integrated into existing surimi production lines. However, factors including energy consumption, enzyme cost, and scale-up feasibility should be evaluated in future pilot-scale studies before industrial implementation. This study did not evaluate sensory quality, storage stability, or microbiological safety of the softened surimi gels. These aspects are critical for commercial application and should be addressed in future research.

## Figures and Tables

**Table 1:** Effects of different PG additions on the texture of surimi gel.

Group	Hardness/g	Springiness	Cohesiveness	Chewiness/g
0% PG	607.40 ± 10.26 ^a^	0.99 ± 0.01 ^a^	0.88 ± 0.00 ^a^	528.44 ± 9.66 ^a^
0.1% PG	391.78 ± 3.84 ^b^	0.92 ± 0.01 ^cd^	0.84 ± 0.01 ^b^	303.53 ± 5.82 ^b^
0.2% PG	244.60 ± 3.65 ^c^	0.94 ± 0.01 ^b^	0.85 ± 0.01 ^a^	204.94 ± 5.49 ^c^
0.3% PG	175.67 ± 1.00 ^d^	0.93 ± 0.01 ^bc^	0.85 ± 0.01 ^b^	139.41 ± 0.77 ^e^
0.4% PG	236.62 ± 3.54 ^c^	0.92 ± 0.01 ^cd^	0.82 ± 0.01 ^d^	177.81 ± 3.23 ^d^
0.5% PG	242.89 ± 1.99 ^c^	0.91 ± 0.00 ^d^	0.80 ± 0.02 ^d^	177.05 ± 3.03 ^d^

Note: Different PG additions were designated as 0%, 0.1%, 0.2%, 0.3%, 0.4%, and 0.5%, the same as below. Different letters (a–e) indicate significant differences among different treatments (*p* < 0.05).

**Table 2:** Analysis of peptide content in the final products of surimi gel digestion treated by ultrasound and enzyme.

Proportion of Molecular Weight %													
Peptide (Da)	<500	<2000	<5000	<10,000	>10,000	5000–10,000	2000–5000	1000–2000	500–1000	300–500	200–300	100–200	<100
NP78	22.71	69.45	92.97	99.47	0.53	6.50	23.52	23.84	22.90	4.55	13.20	2.86	2.10
NP85	25.04	73.98	95.18	99.76	0.24	4.57	21.21	24.31	24.63	4.95	15.09	3.11	1.90
UNP85	27.45	77.12	96.33	99.82	0.18	3.50	19.21	23.91	25.76	5.17	16.68	3.41	2.19
PG85	25.31	73.76	95.39	99.83	0.17	4.44	21.64	24.38	24.07	5.15	14.78	3.04	2.34
UPG85	25.54	74.48	95.76	99.87	0.13	4.11	21.28	24.52	24.42	5.21	15.06	3.09	2.17

Note: NP78 denotes a gel with 78% moisture content, while NP85 and PG85 signify 85% moisture content in the absence and presence of PG treatment, respectively. The surimi gels from the NP85 and PG85 groups underwent ultrasonic treatment prior to the low-temperature reaction, designated as UNP85 and UPG85, respectively.

**Table 3:** Changes of free amino acid content in the final product of ultrasonic and enzyme treated surimi gel digestion.

Amino Acid Content (mg/mL)	NP78	NP85	UNP85	PG85	UPG85
Valine	0.3 ± 0.08 ^a^	0.25 ± 0.01 ^a^	0.29 ± 0.01 ^a^	0.23 ± 0.01 ^a^	0.27 ± 0.04 ^a^
Methionine	0.44 ± 0.24 ^a^	0.23 ± 0.01 ^a^	0.2 ± 0.02 ^a^	0.25 ± 0.03 ^a^	0.31 ± 0.04 ^a^
Leucine	1.05 ± 0.09 ^a^	0.86 ± 0.07 ^b^	0.82 ± 0.04 ^b^	0.89 ± 0.04 ^b^	1.05 ± 0.11 ^a^
Phenylalanine	1.92 ± 0.17 ^a^	1.77 ± 0.16 ^a^	1.58 ± 0.04 ^a^	1.69 ± 0.15 ^a^	1.99 ± 0.37 ^a^
Lysine	2.47 ± 1.87 ^b^	2.69 ± 0.50 ^b^	2.61 ± 0.4 ^b^	3.25 ± 0.35 ^b^	5.17 ± 0.23 ^a^
Glutamic acid	1.36 ± 0.17 ^a^	1.04 ± 0.11 ^c^	1.07 ± 0.04 ^bc^	1.13 ± 0.04 ^abc^	1.3 ± 0.16 ^ab^
Serine	0.63 ± 0.05 ^ab^	0.52 ± 0.06 ^c^	0.55 ± 0.01 ^bc^	0.64 ± 0.04 ^ab^	0.67 ± 0.06 ^a^
Arginine	1.99 ± 0.13 ^a^	1.47 ± 0.09 ^b^	1.55 ± 0.06 ^b^	1.5 ± 0.04 ^b^	1.85 ± 0.21 ^a^
Glycine	1.16 ± 0.23 ^a^	0.89 ± 0.06 ^a^	0.91 ± 0.07 ^a^	0.94 ± 0.04 ^a^	1.03 ± 0.16 ^a^
Alanine	0.59 ± 0.02 ^ab^	0.45 ± 0.05 ^cd^	0.44 ± 0.02 ^d^	0.52 ± 0.03 ^bc^	0.64 ± 0.07 ^a^
Tyrosine	0.59 ± 0.02 ^b^	0.45 ± 0.05 ^bc^	0.44 ± 0.02 ^c^	0.52 ± 0.03 ^bc^	0.64 ± 0.07 ^a^
Total amino acid content	12.59 ± 1.42 ^b^	10.59 ± 1.13 ^b^	10.36 ± 0.50 ^b^	11.5 ± 0.91 ^b^	15.3 ± 1.41 ^a^
Essential amino acid content	6.18 ± 1.58 ^b^	5.8 ± 0.68 ^b^	5.5 ± 0.46 ^b^	6.31 ± 0.56 ^b^	8.78 ± 0.77 ^a^
Ratio of essential amino acids (%)	48.54 ± 7.54 ^b^	54.76 ± 0.86 ^ab^	53.06 ± 2.07 ^ab^	54.84 ± 0.55 ^ab^	57.42 ± 0.45 ^a^

Note: NP78 denotes a gel with 78% moisture content, while NP85 and PG85 signify 85% moisture content in the absence and presence of PG treatment, respectively. The surimi gels from the NP85 and PG85 groups underwent ultrasonic treatment prior to the low-temperature reaction, designated as UNP85 and UPG85, respectively. Different letters (a–d) indicate significant differences among different treatments (*p* < 0.05).

## Data Availability

The original contributions presented in the study are included in the article, further inquiries can be directed to the corresponding author.

## Supplementary Materials

The following supporting information can be downloaded at: https://www.mdpi.com/article/10.3390/foods15173036/s1, Figure S1: Effects of ultrasonic time, power, and low-temperature reaction time on the water holding capacity, cooking loss, gel strength, and whiteness of surimi soft gel; Figure S2: Response surface plots showing the interactive effects of ultrasonic time, power, and enzyme reaction time on the softening degree of surimi gel; Table S1: Factors and levels of the Plackett-Burman design; Table S2: Effect of ultrasonic time on texture properties of softened surimi gel; Table S3: Effect of ultrasonic power on texture properties of softened surimi gel; Table S4: Effect of enzyme reaction time on texture properties of softened surimi gel; Table S5: Response surface optimization of ultrasonic assisted enzyme treatment of surimi gel; Table S6: Plackett-Burman experimental design and results.
