# Supplementary material for: Ultrasound-Assisted Enzymatic Deamidation Treatment for Developing Soft Surimi Gel for Dysphagia: Gel Properties, In Vitro Digestibility, and Protein Mechanisms
Source: Foods. 2026 Aug 28;15(17):3036. doi: 10.3390/foods15173036 (PMC13564660; doi:10.3390/foods15173036)
Supplement: Supplementary file 1 [file foods-15-03036-s001.zip › foods-4454307-Supplementary Materials.pdf]

foods

MDPI

Ultrasound-Assisted Enzymatic Deamidation Treatment for Developing Soft Surimi Gel for Dysphagia: Gel Properties, In Vitro Digestibility, and Protein Mechanisms

Wei Wang, Qing Shao, Lifei Wang, Shumin Yi *, Xuepeng Li, Jianrong Li, Beibei Ye, Chang Zhang, Yongxia Xu and Wenhui Zhu

National & Local Joint Engineering Research Center of Storage, Processing and Safety Control Technology for Fresh Agricultural and Aquatic Products, College of Food Science and Engineering, Bohai University, Jinzhou 121013, China; wwll812002@163.com (W.W.); shaoqing1939827103@163.com (Q.S.); 18641692602@163.com (L.W.); xuepengli8234@163.com (X.L.); 13591278928@163.com (J.L.); beibeiy1993@163.com (B.Y.); zchangzc@163.com (C.Z.); xuyx1009@126.com (Y.X.); wenhuiby130@163.com (W.Z.)

* Correspondence: yishumin@163.com

1. Method

1.1. Single Factor Experimental Group

Ultrasonic power was set at 240 W, with a low-temperature reaction duration of 1 hour, to investigate the influence of varying ultrasonic durations (0 min, 10 min, 20 min, 30 min, 40 min) on the soft gel characteristics of surimi, including TPA, whiteness, cooking loss, and water retention; ultrasonic duration was fixed at 30 min, and low-temperature reaction time was maintained at 1 hour to examine the impact of different ultrasonic power levels (0 W, 160 W, 240 W, 320 W, 400 W) on the soft gel properties of surimi. The impact of varying ultrasonic power levels (0 W, 160 W, 240 W, 320 W, 400 W) on the gel characteristics, texture profile analysis (TPA), whiteness, cooking loss, and water retention of surimi was examined. Additionally, the effects of a specific ultrasonic power (240 W) and ultrasonic duration (30 min), along with low-temperature reaction times (0 min, 30 min, 60 min, 90 min, 120 min), on the same properties of surimi were also analyzed.

1.2. Response Surface Optimization

Based on single-factor experiments, hardness served as the response variable for the identification of key factors, and the values of the combination design levels were recorded and encoded in Table S1.

Table S1. Factors and levels of Plackett–Burman design.

Factors		Levels		
		-1	0	1
Ultrasonic time（min）	A	20	30	40
Ultrasonic power（W）	B	160	240	320
low-temperature reaction time（min）	C	30	60	90

2. Results and Discussion

2.1. Impact of Ultrasonic Treatment Duration on Surimi Soft Gel

Table S2 illustrates that the duration of ultrasound treatment was inversely correlated with the alteration in surimi gel hardness, which attained its minimum at 30 minutes of ultrasound exposure, resulting in a hardness reduction of 45.95% compared with the NPG group and 30.22% compared with the U10 min group. The reduction in hardness

 https://doi.org/10.3390/foods15173036

was ascribed to the cavitation effect of ultrasound, which facilitated the complete unfolding of myofibrillar proteins, thereby exposing additional glutamine residues and augmenting the deamidation efficiency of the PG enzyme. The enhancement in hardness with extended sonication duration may result from the fact that, over a specific timeframe, sonication facilitates the complete unfolding of the protein structure, which, in turn, fosters the comprehensive cross-linking of the protein network during subsequent heat-induced gelation. Prolonged sonication time hindered the enzyme's ability to bind to all substrates within a specified duration, resulting in crosslinking of partially unfolded protein structures during heat-induced gelation [1].

Table S2. Effect of ultrasonic time on texture properties of softened surimi gel.

Group	Hardness/g	Springiness	Cohesiveness	Chewiness /g
NPG	396.24±17.25^{a}	0.93±0.03^{a}	0.84±0.00^{a}	308.33±21.71^{a}
NU	306.88±9.33^{b}	0.97±0.04^{a}	0.84±0.01^{a}	249.90±15.47^{b}
U10 min	307.67±1.52^{b}	0.95±0.03^{a}	0.85±0.00^{a}	248.66±9.83^{b}
U20 min	293.57±11.55^{b}	0.94±0.04^{a}	0.83±0.01^{ab}	230.66±18.67^{b}
U30 min	214.15±14.51^{d}	0.84±0.02^{b}	0.73±0.04^{d}	130.68±13.01^{e}
U40 min	261.80±2.76^{c}	0.92±0.02^{a}	0.80±0.01^{b}	193.87±5.01^{d}

Note: Different letters (a–d) indicate significant differences among different treatments ($p < 0.05$).

Figure S1A illustrates that the water-holding capacity of the surimi gel network augmented with prolonged sonication time, attaining a peak value of 76.78% at 30 minutes of sonication. Cooking loss exhibited an entirely contrary trend, progressively diminishing with increased ultrasonication duration, ultimately attaining a minimum value of 6.42% at 30 minutes of ultrasonication. Water-holding capacity and cooking loss reached their minimum values in the same group, indicating that the water-holding capacity of the surimi gel was optimal under these treatment conditions. The structure of myofibrillar proteins was compromised following ultrasonication, as the deamidation treatment enhanced the protein's hydrophilicity and disrupted the hydrophobic interactions caused by the exposure of moderate quantities of hydrophobic groups. Thermally induced gels were created under high humidity conditions and maximized moisture retention within the protein matrix.

https://doi.org/10.3390/foods15173036

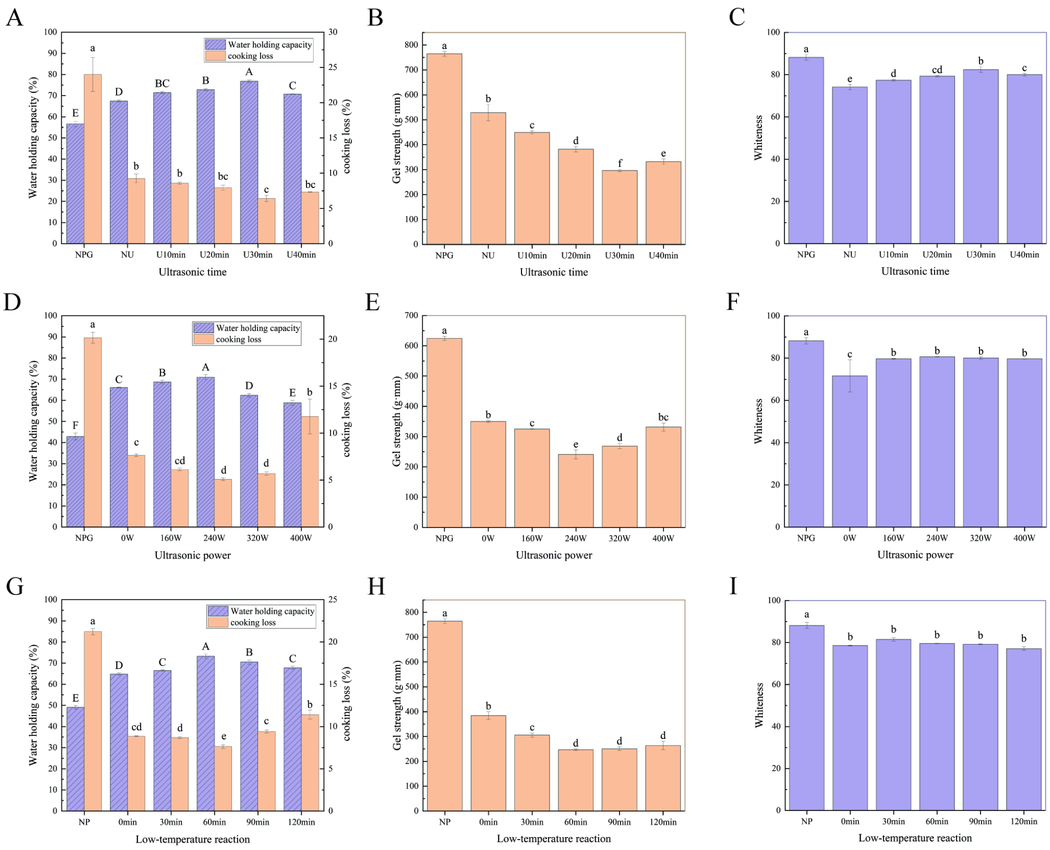

Figure S1. The effects of ultrasonic time on water-holding capacity, cooking loss (A), gel strength (B) and whiteness (C) of surimi soft gel; the effects of ultrasonic power on waterholding capacity, cooking loss (D), gel strength (E) and whiteness (F) of surimi soft gel; the effects of low temperature reaction time on water-holding capacity, cooking loss (G), gel strength (H) and whiteness (I) of surimi soft gel were investigated. Note: Different letters (a–e, A–E) indicate significant differences among different treatments ($p < 0.05$).

The alteration in gel strength exhibited a similar pattern to the variation in hardness (Figure S1 B). The protein network structure was completely unfolded with prolonged sonication time, attributed to the heightened negative charge of the gel network residues resulting from deamidation, which impeded the subsequent formation of numerous disulfide bonds and hydrophobic interactions due to weak interaction forces. The gel strength diminished to its nadir at 30 minutes of sonication, measuring 296.69 g mm, which was markedly inferior to that of the non-enzymatic and enzyme-enzymatic non-sonicated groups. Compared with the NU group, the sonicated group exhibited significant differences in gel strength, texture, and water-holding capacity, thereby enabling the potential for further reduction in the gel properties of surimi gels and the preparation of softer surimi gels.

Figure S1C illustrates that ultrasound-assisted enzyme deamidation treatment can mitigate the reduction in the whiteness value of surimi gel resulting from enzyme addition to a certain degree. Ultrasound-assisted treatment of surimi gel addition enhanced the whiteness of surimi gels, which correlates with alterations in the protein gel network. Generally, the duration of ultrasonication did not significantly influence the whiteness of surimi gel, with changes in whiteness not exceeding 3 units after 20–40 minutes of ultrasonication. At an ultrasonication duration of 30 minutes, the hardness measured at 214.15 ± 14.51 g was significantly lower than that of the other groups ($p < 0.05$), exhibiting the least steaming loss and enhanced water-holding capacity; thus, the 30-minute ultrasonication time was chosen for the subsequent experiment.

2.2. Effect of Ultrasonic Power on Soft Gel

Table S3 illustrates the impact of ultrasound power on the hardness, elasticity, cohesion, and chewiness of softened surimi gel. Overall, ultrasonic power exerted a minimal influence on the elasticity and cohesion of surimi gel, while demonstrating a more pronounced effect on hardness and chewiness. The hardness of surimi gel initially diminished and subsequently augmented with the escalation of ultrasound power. The reduction in hardness was ascribed to ultrasonication at low power, which facilitated the unfolding of the surimi gel structure. The PG enzyme was completely bound to the amino acid residues of the protein gel network; however, ultrasonication further unfolded the protein network, exposing hydrophobic groups and inducing cross-linking within the gel network. High-power ultrasound facilitates more complete unfolding of protein networks, and when weak interactions fail to prevent the formation of strong interactions, the hardness of the surimi gel begins to rise, resulting in an increase in chewiness.

Table S3. Effect of ultrasonic power on texture properties of softened surimi gel.

Group	Hardness/g	Springiness	Cohesiveness	Chewiness /g
NPG	396.24±17.25^{a}	0.93±0.03^{abc}	0.84±0.00^{a}	308.33±21.71^{a}
0 W	258.49±8.06^{bc}	0.99±0.04^{a}	0.83±0.01^{a}	213.86±9.91^{b}
160 W	245.21±5.84^{c}	0.92±0.01^{bc}	0.83±0.01^{a}	186.31±4.62^{b}
240 W	214.09±2.57^{d}	0.87±0.03^{c}	0.78±0.09^{a}	146.17±22.84^{c}
320 W	241.97±5.60^{c}	0.96±0.06^{ab}	0.80±0.02^{a}	186.45±16.64^{b}
400 W	264.42±9.21^{b}	0.97±0.03^{ab}	0.84±0.00^{a}	216.07±13.21^{b}

Note: Different letters (a–d) indicate significant differences among different treatments ($p < 0.05$).

Figure S1D illustrates that the alterations in water-holding capacity and cooking loss of surimi gel exhibited contrasting trends with the escalation of ultrasound power. The water-holding capacity of surimi gel initially increased, followed by a decrease, while the cooking loss first diminished and subsequently escalated. At a power level of 240 W, the gel's water-holding capacity was 70.90%, and the cooking loss was 5.09%. This suggests that as ultrasound power increases, the myofibrillar protein network is completely opened, providing ample substrate for the PG enzyme.

Figure S1E shows a significant effect of ultrasound power on the gel strength of surimi gels ($p < 0.05$). Ultrasound power of 160 W resulted in a reduction in surimi gel strength from 349.94 g mm to 325.16 g mm. Upon further augmentation of the ultrasound power, the gel strength attained its nadir at 240 W, measuring 241.2582 g mm. As a result of the complete expansion of the protein gel network, the enzyme interacted with the substrate more readily, leading to a reduction in gel strength. The weak chemical forces linking the gel network more effectively facilitated the enzyme-substrate interaction. Although the enzymatic reaction exhibited minimal impact on gel strength within a specific time frame, the completely unfolded gel network formed stronger crosslinks, thereby enhancing the strength of surimi gels [2].

Figure S1F illustrates that the ultrasound power enhanced the whiteness of surimi soft gels. Applying a 160 W ultrasound treatment to the soft gel increased whiteness to 79.69%, after which it exhibited minimal variation. Furthermore, there was no statistically significant difference in the whiteness of surimi gels across the 160 W, 240 W, 320 W, and 400 W treatment groups ($p > 0.05$). Sonic treatment enhanced the whiteness of surimi gels, whereas the incorporation of OG marginally reduced the whiteness. The incorporation of

oat β-glucan marginally reduced whiteness, whereas ultrasonic treatment enhanced the whiteness of surimi gel. Ultrasonic treatment modified the surimi gel network by creating adequate space to incorporate additional water molecules [3]. The water molecules within these network structures were primarily affixed to the protein surface, resulting in enhanced whiteness through light reflection [4]. The enzyme deamidation treatment enhances water retention but results in reduced whiteness, likely because the deamidation-treated proteins create dense, regular network structures that encapsulate water within the gel matrix.

In conclusion, at an ultrasonic power of 240 W, the hardness was minimized while the water-holding capacity was maximized, with a significant difference compared with other groups ($p < 0.05$). Therefore, an ultrasonic power of 240 W was selected for subsequent experiments.

2.3. The Effect of Enzyme Reaction Time on Soft Gel After Ultrasonic Treatment

Table S4 shows the effects of low-temperature enzyme reaction time on the hardness, elasticity, cohesiveness, and chewiness of softened surimi gel. Overall, the reaction time had a relatively minor effect on elasticity and cohesiveness, but a more significant impact on hardness and chewiness. As the low-temperature enzyme reaction time increased, the hardness of the surimi gel initially decreased and then rose again. A shorter reaction time promoted deamidation modification of surimi proteins, leading to reduced hardness, as the PG enzyme could effectively act on the unfolded protein substrates after ultrasound treatment. However, by further extending the reaction time, continued enzymatic deamidation increased the negative charge on proteins, significantly altering intermolecular forces. When the low-temperature reaction time became too long, excessive cross-linking of protein molecules occurred, compromising the softening effect of the gel, resulting in increased hardness and chewiness of the surimi gel.

Table S4. Effect of enzyme reaction time on texture properties of softened surimi gel.

Group	Hardness/g	Springiness	Cohesiveness	Chewiness /g
NPG	396.24±17.25^{a}	0.93±0.03^{ab}	0.84±0.00^{a}	308.33±21.71^{a}
0 min	285.76±7.63^{b}	0.99±0.04^{a}	0.82±0.00^{a}	232.11±16.64^{b}
30 min	244.32±12.53^{c}	0.99±0.03^{a}	0.83±0.01^{a}	202.42±13.11^{c}
60 min	207.38±1.47^{d}	0.91±0.03^{b}	0.79±0.02^{b}	150.59±3.99^{d}
90 min	225.44±3.86^{cd}	0.97±0.03^{ab}	0.83±0.01^{a}	181.41±7.27^{c}
120 min	233.35±2.48^{c}	0.96±0.04^{ab}	0.83±0.01^{a}	185.82±10.22^{c}

Note: Different letters (a–d) indicate significant differences among different treatments ($p < 0.05$).

Figure S1G illustrates the impact of reaction time on the textural characteristics of surimi gels following the ultrasonication of surimi at low temperatures. The modification of the surimi gel structure through ultrasonication influenced the duration of the subsequent enzymatic reaction. Consequently, the duration of the enzymatic reaction must be modified. Following ultrasonication without enzyme reaction time and direct heating treatment to halt the reaction, the gel's hardness measured 285.76 g, markedly lower than that of the corresponding unenzymed control group, attributable to the occurrence of the deamidation reaction during the ultrasonication process ($p > 0.05$). The extension of the cryogenic reaction time resulted in a reduction in gel hardness, potentially attributable to an excessively dense protein gel network. Chewiness displayed a similar pattern, initially decreasing and subsequently increasing with the extension of enzyme reaction duration.

Figure S1H illustrates the water-holding capacity of the surimi gel network, indicating that it increased while cooking loss diminished as a result of the enzymatic reaction. The water-holding capacity peaked at 73.22% after 1 hour of the low-temperature reaction, while the cooking loss was minimized at 7.64%. At 120 minutes, the gel's water-holding capacity diminished, and the cooking loss increased to 11.41%, attributed to excessive cross-linking of the gel network resulting from protein network unfolding and enzymatic overreaction.

Figure S1I illustrates the impact of low-temperature reaction duration on the strength of surimi gels. Upon completion of sonication, the surimi gel strength diminished to 384.45 g mm, representing a 49.72% reduction compared with the corresponding non-enzymatic control gel from the same batch. As the enzyme reaction time elapsed, the gel strength consistently diminished, reaching 246.76 g mm at 60 minutes. No significant difference was observed in the subsequent alterations in gel strength ($p > 0.05$). The advancement of enzyme reaction time influenced the whiteness of surimi gel; however, the difference was not statistically significant ($p > 0.05$).

The low-temperature reaction time had less influence on the gel characteristics of surimi soft gel than ultrasonic duration and power. At a low temperature reaction time of 60 minutes, the hardness was minimized, and the water-holding capacity was maximized, with significant differences observed compared with other groups ($p < 0.05$). Consequently, a low temperature reaction time of 60 minutes was selected for subsequent experiments.

2.4. Response Surface Optimization of Ultrasonic-Assisted Treatment Conditions

2.4.1. the Establishment of Regression Model and Anova Analysis

The impact of ultrasound duration, ultrasound intensity, and cryogenic reaction time on the softening degree of surimi gel was examined, as indicated by the one-factor surimi gel hardness results, with the objective of enhancing the softening degree of surimi gel, as presented in Table S5.

Table S5. Response surface optimization of ultrasonic-assisted enzyme treatment of surimi gel. Supplementary figure captions.

	A<br>Ultrasonic time /min	B<br>Ultrasonic power /W	C<br>Low-temperature reaction time /min	Hardness/g
1	1	1	0	300.38
2	-1	0	-1	295.13
3	0	-1	-1	268.94
4	-1	0	1	276.78
5	-1	-1	0	284.29
6	1	0	1	280.70
7	1	-1	0	279.51
8	1	0	-1	270.70
9	0	0	0	227.50
10	0	0	0	221.07
11	0	0	0	234.55

https://doi.org/10.3390/foods15173036

12	-1	1	0	324.91
13	0	-1	1	299.99
14	0	0	0	232.62
15	0	0	0	228.47
16	0	1	1	294.52
17	0	1	-1	322.98

Note: Different letters (a–d) indicate significant differences among different treatments ($p < 0.05$).

The regression model was formulated as follows:

$$Hardness=228.84-6.23A+13.76B-0.72C-4.94AB+7.09AC-14.88BC+26.32A^2+42.10B^2+25.66C^2$$

The ANOVA results presented in Table S6 indicate that the model effect of ultrasonic-assisted enzyme treatment on the softening of surimi gel was highly significant ($p < 0.0001$); R2=0.9801, and the misfit error was not significant ($p > 0.05$), demonstrating that the model was well-fitted. The signal-to-noise ratio was 26.9375, significantly exceeding the critical value of 4, thereby indicating a well-fitted model.

Table S6. Plackett–Burman experimental design and results.

Source	Sum of squares	df	Mean square	F-value	Prob > F	significance
Model	17591.15	9	1954.57	88.46	< 0.0001	significant
A- Ultrasonic time	310.17	1	310.17	14.04	0.0072	**
B- Ultrasonic power	1513.88	1	1513.88	68.51	< 0.0001	**
C- Low-temperature reaction time	4.14	1	4.14	0.1875	0.6781	
AB	97.52	1	97.52	4.41	0.0738	
AC	200.93	1	200.93	9.09	0.0195	*
BC	885.26	1	885.26	40.06	0.0004	**
A^2	2917.62	1	2917.62	132.04	< 0.0001	**
B^2	7463.78	1	7463.78	337.78	< 0.0001	**
C^2	2772.61	1	2772.61	125.48	< 0.0001	**
Residual	154.67	7	22.1			
Lack of Fit	45.53	3	15.18	0.5561	0.6713	not significant
Pure Error	109.15	4	27.29			
Cor Total	17745.83	16				

Note: * indicates significant difference ($p < 0.05$), * * indicates extremely significant difference ($p < 0.01$).

The analysis of the regression equations indicated that the primary terms A ($p < 0.01$) and B ($p < 0.0001$), the interaction term BC ($p < 0.01$), and the quadratic terms A^2 ($p < 0.0001$),

B^2 ($p < 0.0001$), and C^2 ($p < 0.0001$) exerted a highly significant influence on the softening degree of surimi gel; the interaction term AC ($p < 0.05$) demonstrated a significant effect on the softening degree of surimi gel. The effect was substantial.

The F-value and P-value in the ANOVA table indicated that the primary factors affecting the softening degree of surimi gel were ultrasonic power, ultrasonic duration, and enzyme reaction time, with ultrasonic power and duration exerting a significant influence on the response variable.

2.4.2. Analysis and Optimization of Response Surface of Ultrasonic-Assisted Extraction Conditions

Figure S2 illustrates the interplay among the three principal variables: ultrasound duration, ultrasound intensity, and enzyme reaction time, via three-dimensional response surface plots. The impact of the other two variables on the extent of surimi gel softening was examined when either variable was set to zero.

https://doi.org/10.3390/foods15173036

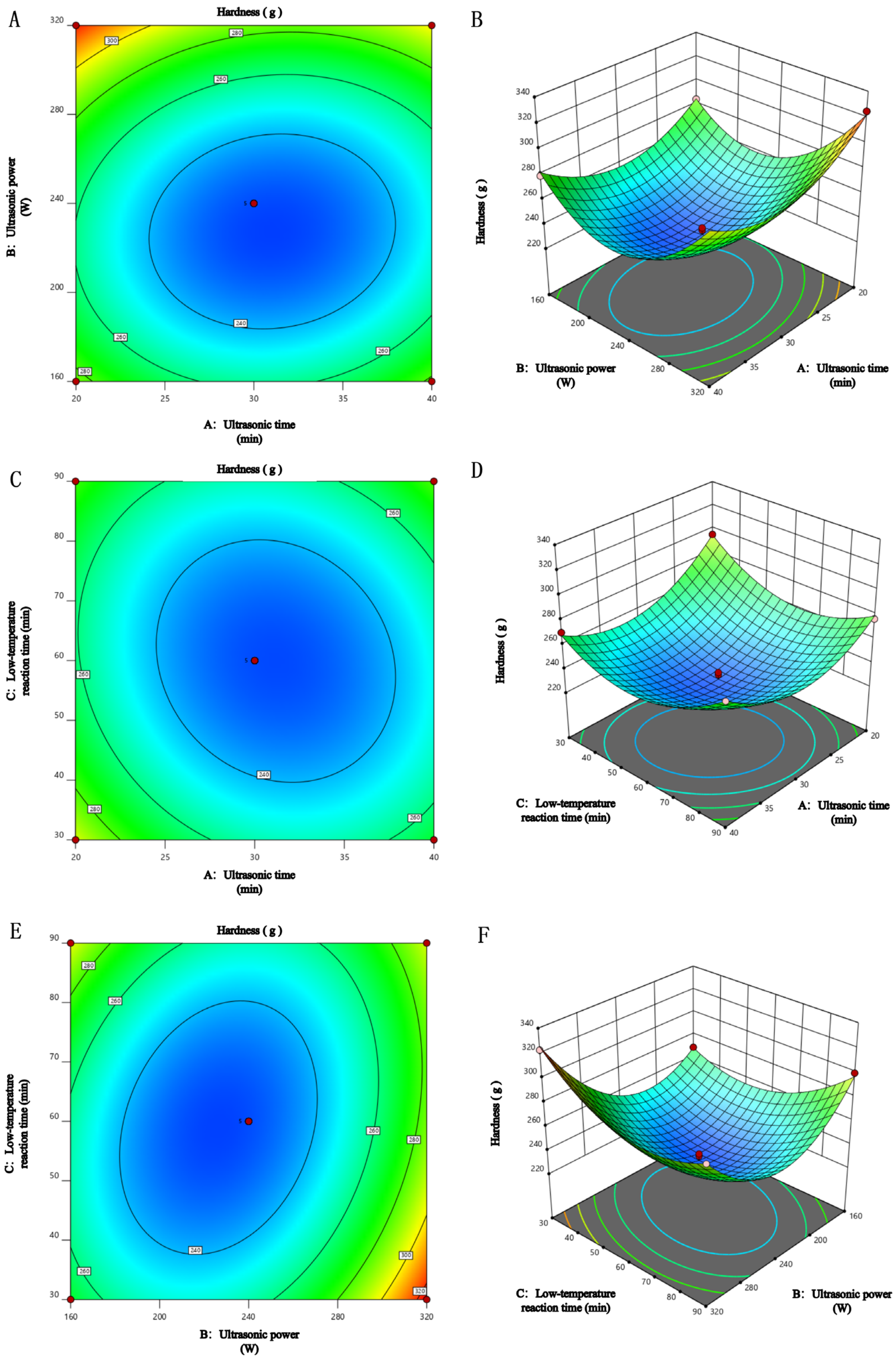

Figure S2. Contour map (A) and response surface map (B) of ultrasonic time and ultrasonic power on the softening degree of surimi gel; contour map (C) and response surface map (D) of ultrasonic time and enzyme reaction time on the degree of surimi gel softening; contour map (E) and response surface map (F) of ultrasonic power and enzyme reaction time on the degree of surimi gel softening).

The analysis of Figures S2A and B indicates that the synergistic effect between ultrasound time and ultrasound power did not achieve statistical significance, with ultrasound power exerting a predominant influence on the gel softening process. The gel hardness

exhibited a nonlinear variation with increasing ultrasonication time and ultrasonic power, initially rising and subsequently declining. The analysis of the interaction effect (Figures S2C and D) reveals a significant synergistic influence of ultrasonication time and low-temperature reaction time, with hardness exhibiting a trend of initial increase followed by a decrease. The curvature of the three-dimensional response surface of E and F in Figure S2 exhibits significant variation, indicating a highly significant interaction effect between low-temperature response time and ultrasound power. The experimental data further corroborate that ultrasound power is a crucial regulator in the gel softening process.

The response surface analysis identified the optimal process parameters for ultrasonic-assisted gel deamidation of surimi as follows: ultrasonic time of 31.09 minutes, ultrasonic power of 226.78 W, and low-temperature reaction time of 58.53 minutes, with a predicted gel hardness of 227.381 grams under these conditions. To facilitate the experiment, the optimal conditions were established as follows: ultrasonic duration of 31 minutes, ultrasonic power of 240 W, and low-temperature reaction duration of 58.53 minutes. To streamline the experimental procedure, the optimal conditions were delineated as follows: ultrasonic duration of 31 minutes, ultrasonic power of 240 W, and a low-temperature reaction time of 59 minutes. The empirical data indicated that the hardness of the optimized gel samples attained 233.51±2.24 g, with a relative deviation from the theoretically predicted value of merely 0.30%. This strongly confirms the reliability of the developed mathematical model, demonstrating that the predicted value of the experimentally derived regression equation closely aligns with the validation value. Furthermore, the established model accurately reflects the real-world scenario, and the derived regression equation holds significant practical relevance for actual production.

References

1. Liu, X.; Sun, X.; Wei, Y.; Ma, Y.; Sun, P.; Li, X. Effects of ultrasonic treatment on physico-chemical properties and structure of tuna (Thunnus tonggol) myofibrillar proteins. *Journal of Food Composition and Analysis* **2022**, *108*, 104438.
2. He, X. L.; Lv, Y. N.; Li, X. P.; Yi, S. M.; Zhao, H. L.; Li, J. R.; Xu, Y. X. Improvement of gelation properties of silver carp surimi through ultrasound-assisted water bath heating. *Ultrasonics Sonochemistry* **2022**, *83*, 105942–105942.
3. He, X. L.; Zhao, H. L.; Xu, Y. X.; Yi, S. M.; Li, J. R.; Li, X. P. Synergistic effects of oat β-glucan combined with ultrasound treatment on gel properties of silver carp surimi. *Ultrasonics Sonochemistry* **2023**, *95*, 106406–106406.
4. Yi, S. M.; Li, Q.; Qiao, C. P.; Zhang, C.; Wang, W.; Xu, Y. X.; Mi, H. B.; Li, X. P.; Li, J. R. Myofibrillar protein conformation enhance gel properties of mixed surimi gels with Nemipterus virgatus and Hypophthalmichthys molitrix. *Food Hydrocolloids* **2020**, *106*.

Disclaimer/Publisher's Note: The statements, opinions and data contained in all publications are solely those of the individual author(s) and contributor(s) and not of MDPI and/or the editor(s). MDPI and/or the editor(s) disclaim responsibility for any injury to people or property resulting from any ideas, methods, instructions or products referred to in the content.

https://doi.org/10.3390/foods15173036
